# Phenylalanine and Tryptophan-Based Surfactants as New Antibacterial Agents: Characterization, Self-Aggregation Properties, and DPPC/Surfactants Vesicles Formulation

**DOI:** 10.3390/pharmaceutics15071856

**Published:** 2023-06-30

**Authors:** Zakaria Hafidi, Lourdes Pérez, Mohammed El Achouri, Ramon Pons

**Affiliations:** 1 Department of Surfactants and Nanobiotechnology, IQAC-CSIC, 08034 Barcelona, Spain; zakaria.hafidi@iqac.csic.es; 2 Laboratoire de Physico-Chimie des Matériaux Inorganiques et Organiques, Centre des Sciences des Matériaux, Ecole Normale Supérieure-Rabat, Mohammed V Université in Rabat, Rabat 5118, Morocco; achmedens@yahoo.fr; 3 Centre des Sciences et Technologies de la Formulation, Rabat 5118, Morocco

**Keywords:** phenylalanine tryptophan, surfactants, antimicrobial activity, vesicles, SAXS

## Abstract

Cationic surfactants based on phenylalanine (C_n_PC_3_NH_3_Cl) and tryptophan (C_n_TC_3_NH_3_Cl) were synthesized using renewable raw materials as starting compounds and a green synthetic procedure. The synthesis, acid-base equilibrium, aggregation properties, and antibacterial activity were investigated. Conductivity and fluorescence were used to establish critical micelle concentrations. Micellization of C_n_PC_3_NH_3_Cl and C_n_TC_3_NH_3_Cl occurred in the ranges of 0.42–16.2 mM and 0.29–4.6 mM, respectively. Since those surfactants have some acidic character, the apparent pK_a_ was determined through titrations, observing increasing acidity with increasing chain length and being slightly more acidic with the phenylalanine than the tryptophan derivatives. Both families showed promising antibacterial efficacy against eight different bacterial strains. Molecular docking studies against the enzyme peptidoglycan glycosyltransferase (PDB ID:2OQO) were used to investigate the potential binding mechanism of target surfactant molecules. According to small angle X-ray scattering (SAXS) results, the surfactants incorporate into DPPC (Dipalmitoyl Phosphatidyl Choline) bilayers without strong perturbation up to high surfactant concentration. Some of the C_12_TC_3_NH_3_Cl/DPPC formulations (40%/60% and 20%/80% molar ratios) exhibited good antibacterial activity, while the others were not effective against the tested bacteria. The strong affinity between DPPC and surfactant molecules, as determined by the DFT (density functional theory) method, could be one of the reasons for the loss of antibacterial activity of these cationic surfactants when they are incorporated in vesicles.

## 1. Introduction

Infectious illnesses are a major problem for both human and animal health, along with the macroeconomy implications [1]. The fast emergence of highly resistant microorganisms, including bacteria, fungi, viruses, and parasites, as a result of the widespread use of antibiotics and biocides during the last century, is jeopardizing our capacity to cure common diseases. Infections produced by multidrug-resistant bacteria are one of the top three dangers to world health, according to the World Health Organization (WHO), and are expected to kill 10 million people per year by 2050 if no action is done [2]. The 68th World Health Assembly endorsed the Global Action Plan on Antimicrobial Resistance [3] in May 2015, with member nations suggesting specific initiatives to address the issue. They include encouraging research on new antimicrobial medicines that can reduce the spread of infectious illnesses.

It has been shown that traditional antimicrobials rapidly lose efficiency owing to microbial resistance, and biocidal diffusion can cause environmental pollution and human toxicity [4]. Vancomycin-resistant enterococci and methicillin-resistant Staphylococcus aureus may live for a day on hospital items, whereas other organisms can survive for 90 days [5]. In this regard, a possible approach to reduce microbial contamination can be the engineering of antimicrobial surfaces by using active coatings [6,7,8].

Surfactants and detergents, which remove debris from surfaces, are advised as a component of medical cleaning and sterilization procedures to perform in tandem with some other antibacterial compounds [9]. In addition, cationic surfactants exhibit exceptional antimicrobial activity, which gives them great potential for use in biological applications. Despite this, the conventional cationic surfactants, based on quaternary ammonium groups, have several environmental problems due to their high level of stability, toxicity, bioaccumulation, and environmental leaching [10,11]. These drawbacks call for the use of new chemicals that meet safety and efficacy requirements. The priority of novel cationic surfactant research and development has turned toward multifunctional molecules that meet legal requirements for human health and environmental protection [12]. 

In this context, amino acid-based surfactants represent a promising starting point in the development of new green and sustainable antimicrobial materials. These are structurally heterogeneous amphiphilic compounds with an amino acid hydrophilic group and an aliphatic chain as the lipophilic moiety. Due to their amphiphilic nature, positive charge, and high surface activity, these cationic amphiphiles, like QACs (Quaternary Ammonium Compounds), are expected to act via perturbation of the bacterial membrane [13,14,15]. This mode of action hampers the development of resistant bacteria. Moreover, in contrast with QACs, given that cationic amphiphiles can be designed to be biodegradable in the environment, they are unlikely to induce bacterial resistance through accumulation in the environment. 

Green amino acid surfactants are promising candidates for preparing cationic vesicles. These vesicles are interesting aggregates that can be used in numerous biomedical applications [16]. They can act as antimicrobial formulations. They can encapsulate polar drugs in their aqueous compartment and hydrophobic compounds can be incorporated into the hydrocarbon domain. Moreover, due to their cationic character, they interact with the negatively charged DNA to form lipoplexes that can be used as vectors in gene therapy [17]. Usually, quaternary ammonium bromide surfactants (QACs) are chosen as the positively charged components [18,19]. However, the drawbacks of these compounds preclude the use of these systems in some biomedical applications. 

**Figure 1:**
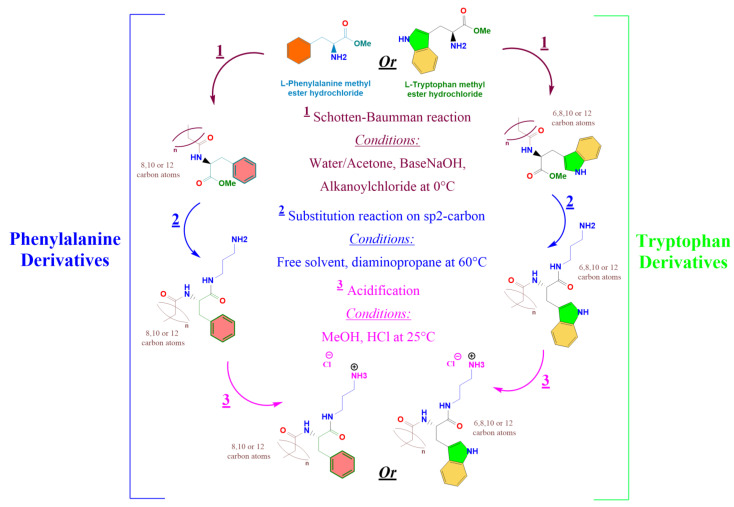
Design strategy of new antimicrobial cationic surfactants based on tryptophan and phenylalanine. (*n* = 6 C_8_TC_3_NH_3_Cl, n = 8 C_10_TC_3_NH_3_Cl and C_10_PC_3_NH_3_Cl, *n* = 10 C_12_TC_3_NH_3_Cl and C_12_PC_3_NH_3_Cl and *n* = 12 C_14_TC_3_NH_3_Cl_,_ and C_14_PC_3_NH_3_ Cl).

In this work, we have designed and synthesized two new families of cationic phenylalanine and tryptophan-based surfactants that can be prepared using a synthetic procedure that agree with the green chemistry principles. The chemical structure of these new surfactants was planned considering the structural requirements for readily biodegradable surfactants (Figure 1). The micellization properties were studied using conductivity, fluorescence, and SAXS measurements. Because the cationic character of these surfactants is based on protonated amino groups, the dissociation constant has been determined. We have studied mixtures with DPPC to prepare cationic vesicles. The antimicrobial effectivity of both the pure surfactants and their liposomal formulations was determined. To rationalize the observed biological behavior, we have characterized the mixed systems by using SAXS and using DFT calculations. Moreover, molecular docking was implemented to study the interaction of these surfactants with the enzyme peptidoglycan glycosyltransferase (PBD ID:2OQO).

## 2. Materials and Methods

All solvents were reagent grade and used without further purification. Octanoyl chloride, Caproyl chloride, Lauryl chloride, and Myristoyl chloride were obtained from Fluka, and amino acids from TCI Europe. Trifluoroacetic acid (TFA) and dimethylformamide (DMF) were supplied by Merck. The culture media Sabouraud dextrose agar and CHROMagar were acquired from Himedia (Mumbai, India). The ATCC^®^ Medium 712: PYG w/Additives was from Manassas. 

### 2.1. Synthesis

#### 2.1.1. Preparation of N^α^-acyl-tryptophan Methyl Ester (C_n_TOM) and N^α^-acyl-phenylalanine Methyl Ester (C_n_POM) (Figure 1)

L-phenylalanine methyl ester or L-tryptophan methyl ester (5 g, 0.02 mol) were dissolved in 250 mL of acetone/water (34/60). The pH of the mixture was adjusted to 10 using an aqueous NaOH solution (1 M). After, 6.12 g (1.3 × 0.02 mol) of alkanoyl chloride (Octanoyl, Decanoyl, Lauroyl, or Myristoyl) was added dropwise, and the mixture was stirred at room temperature for 4 h. The solid product obtained was filtered, washed with a solution of acetone/water at pH = 10, and dried using freeze-drying. Pure compounds were obtained by several crystallizations from hot acetonitrile. These intermediates were identified using HPLC, MS spectroscopy, and NMR.

#### 2.1.2. Preparation of the Target Surfactants (C_n_PC_3_NH_3_Cl) and (C_n_TC_3_NH_3_Cl)

C**_n_**POM or C**_n_**TOM (1 g, 2 mmol) was weighed in a round flask and 2.06 g (20 mmol) of 1,3-diaminopropane was added. The reaction mixture was maintained at 60 °C for 2 h. C_n_PNH_2_ and C_n_TNH_2_ were obtained by removing diamine under reduced pressure. Then, C_n_PNH_2_ or C_n_TNH_2_ were reacted with HCl in a methanol medium to obtain the target C_n_TC_3_NH_3_Cl and C_n_PC_3_NH_3_Cl. The reaction mixture was maintained at room temperature for 30 min, the excess HCl was removed with a rotary evaporator, and the sample was dried overnight under freeze-drying. The target surfactants were identified using HPLC, MS, and NMR (see Electronic Supplementary Information).

### 2.2. HPLC Analysis

The reaction progress, as well as the purity of all prepared molecules, were monitored with an HPLC (Merck-Hitachi D-2500) using a UV–vis detector (L- 4250 at 215 nm) and a Lichrospher 100 CN. The gradient elution profile used went from 25 %B to 95 %B in 24 min. Aqueous phase A was 0.1% (vol/vol) trifluoro acetic acid (TFA) in H_2_O, and organic phase B was 0.085% (*v*/*v*) of TFA in H_2_O/CH_3_CN (1:4).

### 2.3. NMR Experiments 

Sample solutions were prepared in deuterated methanol (20 mg/0.8 mL) using 5 mm NMR tubes. ^1^H NMR and ^13^C NMR analyses were recorded on a Varian 400 MHz spectrometer. All experiments were performed in an indirect broadband probe. Data were processed with the MestReNova (Mestrelab Research 14.1) software.

### 2.4. Mass Spectroscopy 

Surfactant solutions (in the 1 × 10^−4^ to 1 × 10^−6^ M range) were prepared in methanol. High-resolution mass spectra (HRMS) were performed on Acquity UPLC System and an LCT PremierTM XE Benchtop orthogonal acceleration time-of-flight (Waters Corporation, Milford, MA, USA) equipped with an electrospray ionization source. All data were processed and displayed using MestReNova (Mestrelab Research 14.1) software.

### 2.5. Fluorescence Measurements

Steady-state fluorescence measurements of pyrene in surfactant solutions were performed at 25.0 ± 0.1 °C (Shidmadzu RF 540 spectrofluorometer, Kyoto, Japan) using a 1 cm path-length quartz cuvette. From a stock solution of pure surfactants prepared in a pyrene-deionized aqueous solution of 10^−6^ M, different dilutions were prepared. The emission spectrum was recorded from 340 to 450 nm using an excitation wavelength of 332 nm. Excitation and emission band slits were kept at 2 nm. The ratio of the first to the third vibrionic peaks (I_1_/I_3_) in the emission spectra of pyrene depends on the environment polarity and can be used to calculate the CMC [20].

### 2.6. Conductivity Measurements

Conductivity experiments were performed at 25.0 ± 0.1 °C using a Thermo Orion 5 Star multiparameter instrument (Waltham, MA, USA) equipped with an Orion conductivity cell 913005MD with an epoxy/graphite electrode and a cell constant of 0.475 cm^−1^. Samples for conductivity measurements were prepared in Millipore ultra-pure water (Burlington, MA, USA). Measurements were made at increasing concentrations to reduce errors from possible electrode contamination. The conductivity of water was subtracted from the measured conductivity of each sample. All measurements were performed in triplicate.

### 2.7. Determination of pK_a_

The apparent pKa values of the tryptophan and phenylalanine-based surfactants were determined by titration of about 30 µmol of aqueous surfactant solution at around 2 mM concentration with 20 mM aqueous sodium hydroxide at 25 °C. Because of high pK_a_ values, back-titration was also performed. In the cases where both procedures show an equivalence point, the obtained values for pK_a_ were coincident within 0.3 units in the worst case. pH was measured using a pH electrode model 8103BN ROSS semi-micro ThermoOrion (Columbia, MD, USA) connected to a DualStar ThermoOrion pH-meter. Titrations were performed under a nitrogen gas atmosphere with constant magnetic stirring. The apparent pK_a_ was determined based on the pH corresponding to the semi-equivalence point of the neutralization curve.

### 2.8. Small X-ray Scattering (SAXS)

Small-Angle X-ray Scattering (SAXS) measurements were used to gain structural information on the aggregation of the surfactants and their mixtures with phospholipids. Measurements were carried out using an S3-MICRO (Hecus X-ray Systems GmbH, Graz, Austria) coupled to a GENIX-Fox 3D X-ray source (Xenocs, Grenoble, France) working at 50 kV and 1 mA. The detector-focused X-ray beam is produced by a cooper anode with λ = 0.1542 nm Cu K_α_ line with more than 97% purity and less than 0.3% K_β_. A position X-ray detector was used in a transmission configuration (PSD-50 detector, Hecus X-ray Systems GmbH, Graz, Austria). The temperature was controlled by means of a TCCS-3 Peltier (Hecus X-ray Systems GmbH, Graz, Austria), and the scattering patterns were recorded at 25 °C. The dispersions were loaded in a flow-through glass capillary of 1 mm diameter and 10 μm wall thickness (Hilgenberg, Germany). The SAXS scattering curves are shown as a function of the scattering vector modulus, according to the following formula equation (Equation (1)):

(1) $$q=\left(\frac{4\pi }{\lambda }\right)*\sin\left(\frac{\theta }{2}\right)$$

where *θ* is the scattering angle and λ the wavelength. The *q* values with this setup ranged from 0.2 to 6 nm^−1^. The scattering vector was calibrated by measuring a standard silver behenate sample. Scattering was accumulated in 20 min files for a total of 2 h. Once verified that there is no drift in the measurements, those spectra are summed and the resultant is background subtracted (water in this case) and scaled to absolute intensity by comparison with the water intensity. SAXS patterns were analyzed using a home-made fitting procedure based on a Gaussian description of the bilayers and using a Levenberg-Marquardt minimization scheme [21,22,23,24,25,26] and using a sphere or ellipsoid core-shell description for the micellar surfactants.

### 2.9. Antibacterial Activity

Serial dilutions of the surfactant solutions and their vesicles were evaluated for antibacterial activity. The bacterial strains utilized were: *Bacillus subtillis* (ATCC 6633)*, Staphylococcus epidermidis* (ATTC 12228)*, Staphylococcus aureus* (ATCC 6538)*, Listeria manocytogenes* (ATCC 153131)*, Enterococcus faecalis* (ATCC 29212)*, Escherichia coli* (ATCC 8739)*, Acinetobacter baumannii* (ATCC 19606), *and Klebsiella aerogenes* (ATCC 13048). These bacteria were kept and grown following the guidelines set out by the National Council for Clinical Laboratory Standards [27]. The minimal inhibitory concentration (MIC) of the new surfactants was determined using a broth microdilution assay [28]. Serial dilutions of every compound, between 256 and 2 μg/mL, in MH Broth were dispensed (200 μL) in the corresponding wells of a 96-well polypropylene microtiter plate. The nutrient broth starter culture of each bacterial strain (10 μL) was added to achieve the final inoculum of ca. 5 × 10^−5^ colony-forming units (CFU) per mL. Nutrient broth medium without the compound served as growth control. The development of turbidity in an inoculated medium is a function of growth and reflects an increase in both mass and cell number. The MIC was defined as the lowest concentration of antibacterial agent that inhibited the development of visible growth after 24 h of incubation at 37 °C. To confirm this observation, 20 μL of resazurin at 0.015% *w*/*v* was added to each well and left to react for approximately 1 h at 35 °C. After the incubation period, the indication of bacterial growth, i.e., changing from blue to pink, confirmed the MIC value. To obtain the MBC, the antimicrobial concentration corresponding to at least 3 log reductions of viable cells, an aliquot of 10 μL of the MIC well, and the 2 concentrations immediately above were seeded over agar MH and incubated for 24 h at 35 °C. The MBC was determined as the lowest concentration in which no colonies were observed on the agar plates.

### 2.10. Hemolytic Activity 

For the preparation of erythrocyte suspensions, fresh blood was taken from a rabbit using a procedure approved by the institutional ethics committee on animal experimentation. The erythrocytes were washed three times in PBS (pH 7.4). Finally, erythrocytes were suspended in PBS at a cell density of 8 × 10^9^ cells/mL. 

To determine the hemolytic activity of the catanionic mixtures prepared in this work, we adapted the procedure described by Pape et al. [29]. A series of different volumes of a concentrated sample, ranging from 10 to 80 μL, were placed in small Eppendorf tubes containing 25 μL of erythrocyte suspension and PBS was added to each tube to a total volume of 1 mL. Samples were shaken for 10 min at room temperature, and the tubes were then centrifuged (5 min at 10,000 rpm). The percentage of hemolysis was determined by comparing the absorbance (540 nm) of the supernatant of the samples with that of the control totally hemolyzed with distilled water. Concentration-response curves were determined from the hemolysis results and used to calculate the concentration inducing 50% hemolysis (HC_50_).

### 2.11. Vesicles Preparations

Vesicle formulations were prepared by slightly modifying the film hydration method [30]. The surfactants and phospholipid (DPPC) were dissolved in a methanol/chloroform mixture with different Surfactants/DPPC ratios: 80/20, 60/40, 40/60, and 20/80. The organic solvents were evaporated under vacuum, and the resulting lipid film was hydrated with 1 mL of deionized water under sonication (45 min at 40 °C). 

### 2.12. Size Distribution Analysis 

Size distribution of the DPPC/Surfactants vesicles was studied by optical microscopy using a Zeiss (Jena, Germany) light microscope equipped with a Linkam LTS120 hot stage, controlled by a PE94 unit. Images were acquired with a Canon PowerShot S90 Wide Zoom digital camera. Sizing was performed using ImageJ software of calibrated images [31].

### 2.13. Molecular Docking Studies

**Figure 2:**
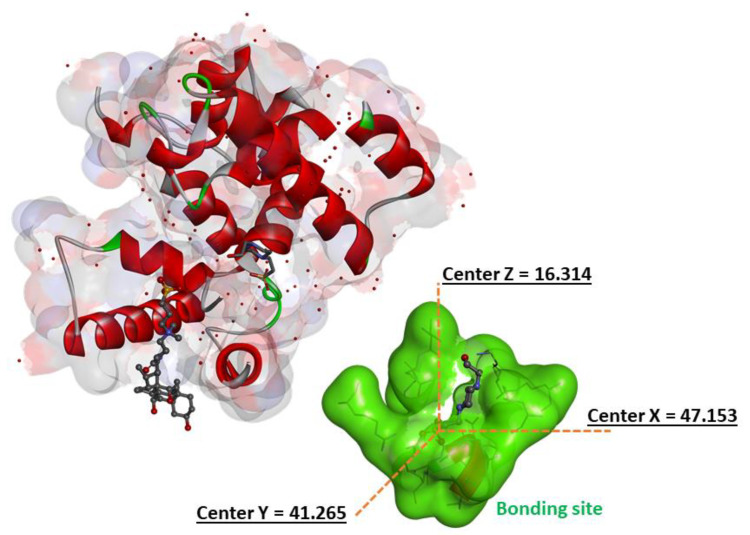
The 3D crystal structure of peptidoglycan glycosyltransferase (PDB ID:2OQO) and the estimated parameters of grid size (x = 47.153, y = 41.265, z = 16.314).

The binding mechanisms of surfactants inside the peptidoglycan glycosyltransferase protein were explored using Autodock Vina molecular docking simulation [32]. The target enzyme was the 3D Crystal structure of a peptidoglycan glycosyltransferase (PDB ID: 2OQO) (Figure 2) [33]. PYMOL [34] was used to remove water molecules, ligands, and other species not included in the protein structure from the target crystal structure (2OQO). Polar hydrogen atoms were introduced to the constructed protein structure to correct ionization and tautomeric states of amino acid residues [35]. AutoGrid was used to prepare the geometry of the binding site by employing a grid box with 14 (20, 62, 24) points that enclose the original ligand (CPS), and the box spacing was 0.37 Å. All parameters were set to their default values in Autodock Tools 1.5.4 44 [32]. Chemdraw12.0 software was used to create all of the surfactant ligands [36]. The geometry of these ligands and Cn benzalkonium derivatives references (From C8 to C14 carbons atoms) was then adjusted using Molecular Force Field to determine the most stable conformation (MMFF94). To enable docking in AutoDock Vina, the ligand and target protein files were converted to PDBQT format. Discovery Studio Client v16 [37] software was used to examine the interactions of complicated protein–ligand conformations.

### 2.14. Quantum Chemical Parameters Calculations

Quantum chemical methods and molecular modeling techniques help to define a large number of molecular descriptors characterizing the reactivity, form, and binding properties of a complete molecule as well as molecular fragments and substituents. The use of theoretical parameters can help to design a theoretical approach that explains the DPPC/surfactant interactions [38]. Computational chemistry (the density functional theory (DFT)) was used as a tool to study the electronic distribution of pure surfactant molecules, as well as their complexes, with DPPC. This approach allows us to calculate and analyze the molecular affinity between these surfactants and the DPPC. All calculations were carried out with GAUSSIAN 09 program [39], and the visualization of the results was performed with Gauss View [39]. In this work, geometry optimization and other calculations were carried out using the Density Functional Theory (DFT) with Becke, 3-parameter, Lee–Yang–Parr (B3LYP) functional. The 6-311g(d) was employed as the basis set for the calculations. Afterward, several quantum chemical parameters of isolated molecules were calculated: energy of highest occupied molecular orbital (E_HOMO_), lowest unoccupied molecular orbital energy (E_LUMO_), and gap energy between E_LUMO_ and E_HOMO_ (ΔE= E_HOMO_ − E_LUMO_). Further, other molecular reactivity descriptors were calculated: electronegativity (χ) and chemical hardness (*η*) of the two molecules, which are given by the following equations [40,41].

(2) $$\chi =\frac{E_{\mathrm{LUMO}}+E_{\mathrm{HOMO}}}{2}$$

(3) $$\eta =\frac{E_{\mathrm{LUMO}}-E_{\mathrm{HOMO}}}{2}$$

## 3. Results

### 3.1. Synthesis

**Figure 3:**
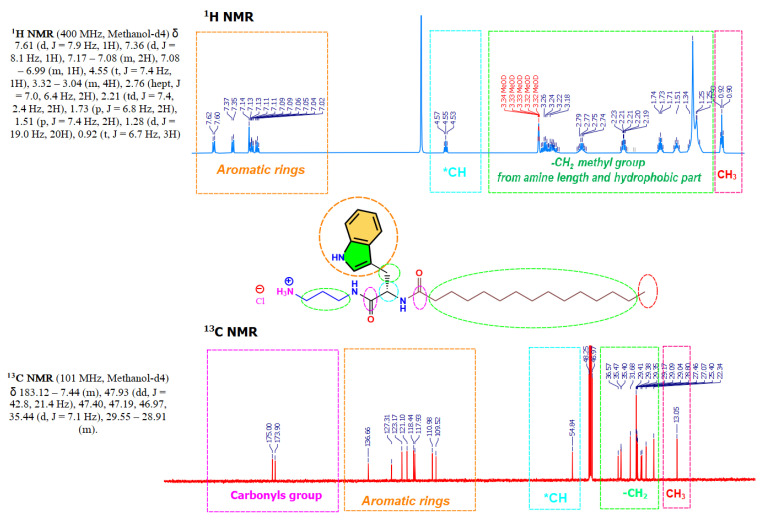
^1^H NMR (CD_3_OD, 400 MHz), and ^13^C NMR (CD_3_OD, 101 MHz) spectra of C_14_TC_3_NH_3_Cl.

The synthesis of these compounds was performed using renewable raw materials such as phenylalanine and tryptophan amino acids and fatty acid chlorides; moreover, the synthetic procedure does not require the use of toxic organic solvents or high temperatures. The surfactant has been designed to be readily biodegradable compounds; in fact, preliminary results showed that, effectively, these surfactants show very good biodegradation levels. The synthesis of N^α^-acyl-tryptophan and N^α^-acyl-Phenylaniline-based surfactants (C_n_TC_3_NH_3_Cl, C_n_PC_3_NH_3_Cl) requires connecting the aliphatic chain to the amino acid. The synthesis was carried out in 2 steps: (a) N-acylation of the α-amino group of the amino acid with the corresponding fatty acid chloride, the reaction progress was followed through HPLC, and after 4 h, the conversion was higher than 90%, and (b) the second step involves the building of an amide bond by condensation of the methyl ester group of the C_n_POM/C_n_TOM with one of the amine groups of the 1,3-diaminopropane, followed by acidification with HCl in a methanolic solution (Figure 1). This synthetic approach is straightforward and fits three essential requirements of green chemistry for organic synthesis: utilization of renewable starting materials and a synthetic procedure consisting of two simple chemical reactions that do not require the use of organic solvent nor protecting amino acids. Moreover, the hydrophobic group is linked to the α-amine group of surfactants through amide bonds to ensure high biodegradability levels and good chemical stability [42]. Compounds were obtained as white powders with yields ranging from 70 to 80%. The purity of the synthesized compounds was greater than 96%, as assessed by mass spectroscopy and ^1^H NMR, ^13^C NMR (see Electronic Supporting Information File). Figure 3 shows a typical example of ^1^H NMR and ^13^C NMR corresponding to C_14_TC_3_NH_3_Cl.

### 3.2. Apparent pKa Values

The cationic charge of the studied surfactants is situated on one protonated amine group; then, in aqueous solutions, these surfactants show an acid–base equilibrium. In the absence of aggregation, the charge of the molecules depends only on the group pKa and the pH of the media. However, in surfactants, we must talk about apparent pKa because the acid–base equilibria depend on the aggregation of the molecules due to several phenomena, such as local concentration and the presence of an interphase or head-group–headgroup interaction [43]. Given that the biological properties of these surfactants are governed by the charge density [44], it is essential to know the number of cationic charges at the pH at which biological activity was determined as well the pK_a_ values. Two distinct equilibriums must be taken into consideration when measuring the apparent pK_a_ of these compounds, monomer–aggregates equilibrium and acid–base equilibrium. Aggregation is known to cause shifts in the acid–base equilibrium and occurs at lower concentrations in neutral species than in charged ones. In micelles, molecule deprotonation is favored because it decreases the electrostatic repulsion among the head groups [45]. 

**Figure 4:**
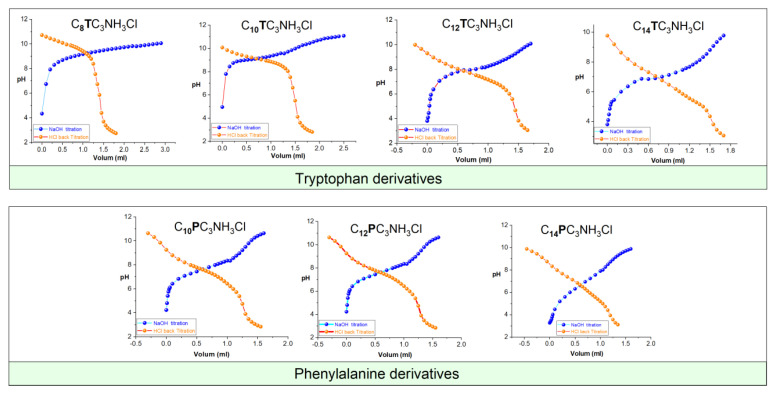
pH as a function of NaOH titration and HCl back-titration volumes 25 °C for C_n_TC_3_NH_3_Cl and C_n_PC_3_NH_3_Cl (these graphs only show the general view of the different molecules; the graphs with higher resolution are presented in the (Electronic Supporting Information File).

The investigated compounds were titrated with NaOH, and the pH values were recorded and plotted as a function of the NaOH volume added (Figure 4). According to the acid–base equilibria, the pK_a_ corresponds to the pH value obtained at the semi-equivalence point of the titration (Table 1). 

The pK_a_ values indicate that, as expected, the density charge of these compounds depends on the pH of the medium, and consequently, they can be considered pH-sensitive surfactants. This behavior is common to all surfactants with the positive charge situated on a protonated amine group [46,47]. The advantage of these types of surfactants is that their physicochemical and biological properties can be modulated by pH. This property makes these compounds interesting candidates to be used in biomedical applications such as gene therapy because they improve the release of DNA [48].

On the one hand, for the same alkyl chain, similar pK_a_ values were obtained (the phenylalanine surfactants being slightly more acidic); on the other hand, for the same head group, it was observed that the pK_a_ decreased as the alkyl chain increased. It seems that with increasing alkyl chain lengths, the hydrophobic chains become more tightly packed; then, to avoid unfavorable repulsions of the positively charged polar heads, the surfactants release more protons, showing lower pK_a_ values. This behavior agrees with that obtained for N^ε^-lysine based surfactants [36] and phenylalanine and that reported by Spelios et al. [49].

The pK_a_ of these surfactants is higher than that reported for amino-acid-based surfactants with the cationic charge located on the α-amine group [42] and agrees with that obtained for lysine-based surfactants with the charge on the ε-amine group [43]. These results suggest that the pK_a_ of the protonated amine groups is greater when the amine group is separated from the other functional groups of the molecules by some CH_2_ groups.

The obtained pK_a_ of the C_8_-C_12_ derivatives are equal to or higher than 7.5. At pH equal to the pK_a_, the compound is 50% protonated, while for pH values 1 unit lower than the pK_a_, it can be considered that the pH-sensitive surfactants are totally protonated. Then, it can be stated that the C_8_-C_12_ derivatives are totally protonated at the pH used to measure the antimicrobial activity, while the C_14_ derivative maintains around 50% of its cationic charges. 

### 3.3. Aggregation Properties

**Figure 5:**
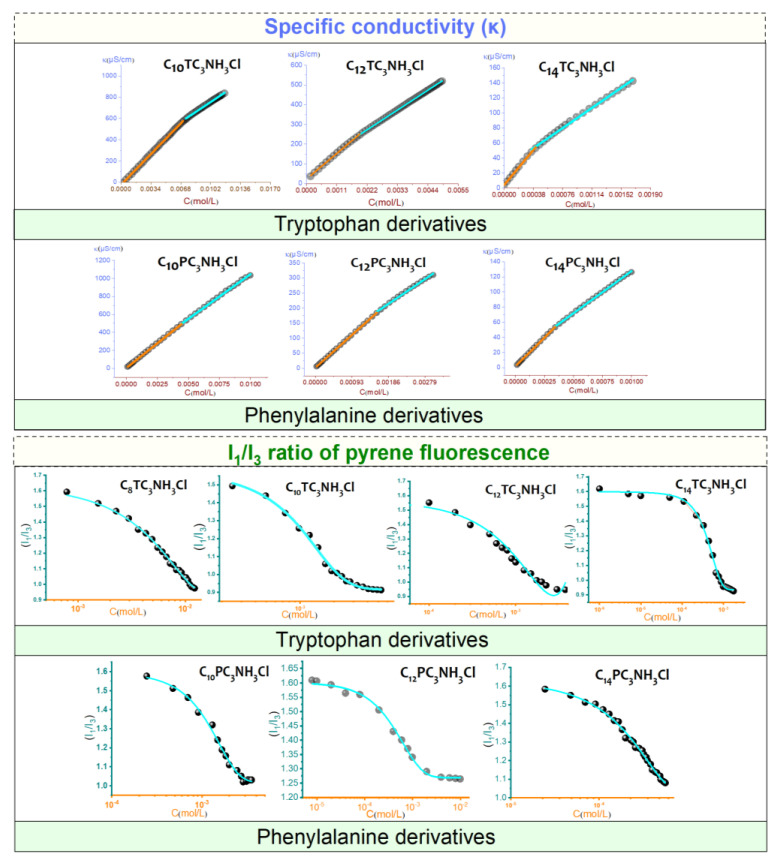
Specific conductivity (κ) and I_1_/I_3_ ratio of pyrene fluorescence as a function of the concentration C_n_TC_3_NH_3_Cl and C_n_PhNH_3_Cl. These graphs only show the general trends; the graphs with higher resolutions are given in the Electronic Supporting Information File.

The electrical conductivity of surfactant solutions changes to different extents below and above the CMC (Figure 5). The hypothesis of aggregation of molecules in clusters beyond a threshold concentration was first proposed by McBain in 1913 [50]. In water, the cationic surfactant molecules form micelles in which the hydrophobic parts cluster together and are protected from contact with water by an envelope of polar heads. Therefore, the graph of conductivity and concentration results in two straight lines that intersect at the CMC.

The CMC and the degree of ionization were determined. The values obtained are listed in Table 1. The degree of ionization (α) was determined from the ratio between the slopes above and below the CMC of the conductivity curves. The degree of counterion binding (β) is defined as β = 1 − α. The degree of binding of the counterion to the micelle depends on its surface charge density. Counterion bindings of about 0.6–0.7 are frequent for cationic surfactants. Obtaining the counterion binding from the difference in slope for pH-sensitive surfactants may be underestimated due to the release of protons associated with increasing concentration of surfactant and especially at the micellization point [43]. The CMC was also investigated by steady-state fluorescence measurements using pyrene as a solvatochromic probe. The intensity ratio between the first (I_1_) and third (I_3_) vibrionic peaks of pyrene in the emission spectra is a polarity index for the microenvironment of the fluorescent probe. 

The figure shows the changes in the I_1_/I_3_ ratio of the emission spectra plotted as a function of surfactant concentration. At the initial concentrations, the ratio was about 1.6, which is comparable to that of pyrene in pure water. At the most dilute concentrations, no significant changes in the I_1_/I_3_ ratio were observed; due to the absence of surfactant aggregates, the polarity of the microenvironment around the pyrene molecules is the same. Then, an abrupt decrease in I_1_/I_3_ intensity is observed, indicating the formation of aggregates and the incorporation of pyrene into the micellar core. The data were fitted to Boltzmann-type sigmoidal curves, and the CMC values were taken as the midpoint of the transitions. In general, the CMC values derived from the fluorescence measurements are of the same order as those determined by conductivity. The I_1_/I_3_ ratio above the CMC was similar for both families of surfactants, suggesting that their micelles had comparable polarity. Note that the preparation of C_14_PC_3_NH_3_Cl at high concentration is complicated by the increase in viscosity observed for these solutions.

The CMC values of the two families studied, N-acyl-tryptophan salts and N-acyl-phenylalanine salts in water, depended on the alkyl chain length. For homologs with the same polar head, the CMC decreases with an increasing number of carbon atoms in the hydrophobic chain. In all cases, the CMC values are of the same order for the different polar headgroups, as expected. More hydrophilic species should be more soluble and, therefore, less prone to form micelles. These CMC values are significantly lower than those of surfactant-based amino acids with a linear hydrocarbon N^α^-alkyl-arginine methyl ester (LAM CMC = 5.8 mM [15]) and N^α^-alkyl-lysine methyl ester (LLM CMC = 7.2 mM [51]). The introduction of aromatic amino acids increases the hydrophobic character of these surfactants and consequently reduces their CMC values. C_n_TC_3_NH_3_Cl showed slightly higher CMC values than the phenylalanine derivatives C_n_PC_3_NH_3_Cl. This behavior can be attributed to steric hindrance; however, since two rings (in the case of tryptophan) have a larger molecular volume than only one ring (in the case of phenylaniline), we would expect the phenylaniline homologs to have bigger CMC than the tryptophan derivatives. Our experimental results contradict this expectation, probably because of the presence of the indole. In a comparative study based on the theoretical calculations of AB initio, Steve Schreiner et al. [52] showed that among a series of amino acids, the aromatic group constituents of amino acids such as tyrosine and tryptophan will prefer to form H-bonds of the conventional sort with a water molecule. The OH^…^N bond involving histidine is the strongest, followed by the OH^…^O bond where tyrosine acts as a donor, and then by the NH^…^O bond of tryptophan. If such bonds are unattainable, or in the case of phenylalanine that contains no heteroatoms, other stabilizing interactions are possible, albeit somewhat weaker. These results suggest that the presence of the indole group in the tryptophan-based surfactants increases their hydrophilic character, which implies an increase in their CMC values compared to that of C_n_PC_3_NH_3_Cl derivatives.

### 3.4. Cationic Vesicles 

**Figure 6:**
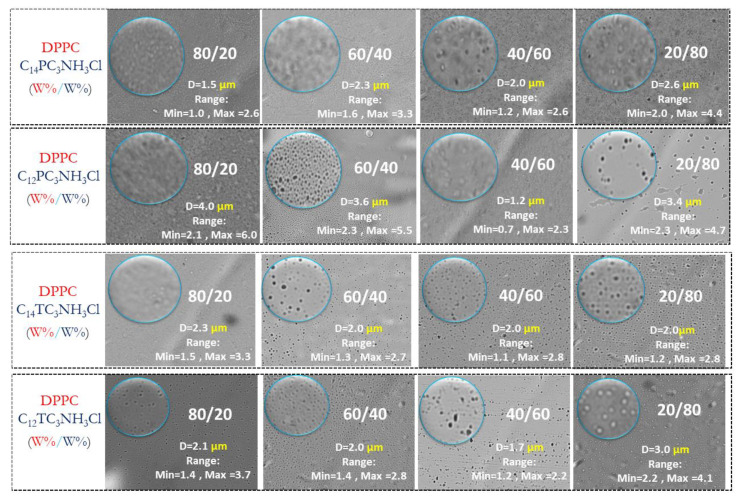
Optical microscopic image of prepared liposomes (40× objective, the width × height of the images corresponds to 274 × 205 µm). The median diameter and the minimum and maximum diameters observed are shown in the pictures.

Phospholipid vesicles (liposomes) are under investigation as models for biological membranes and as carriers for various bioactive agents such as drugs, diagnostic and genetic materials, and vaccines [53]. The four most antimicrobial surfactants (C_14_PC_3_NH_3_Cl, C_12_PC_3_NH_3_Cl, C_14_TC_3_NH_3_Cl, and C_12_TC_3_NH_3_Cl) were chosen to formulate cationic vesicles based on DPPC. Four molar ratios were studied for the mixtures DPPC/Surfactants: 80:20, 60:40, 40:60, and 20:80. Vesicle morphology, shape, and size were monitored by optical microscopy. Figure 6 shows micrograph images from different formulation samples; the field of view is around 274 × 205 µm. A population group of particles is indicated and surrounded by a blue circle. In this area, we performed a statistical analysis of the liposomes and their size distribution using histogram plots fitted with a Gaussian curve. 

According to these results, it can be stated that large vesicles are present in all vesicle formulations. There are not clear trends in the determined sizes; although the median diameters may be significantly different, the ranges of observed diameters largely overlap, blurring possible composition effects. With the observed sizes, the use of light scattering for the determination of sizes and for the determination of z-potential was not possible (although a proper determination of z-potential was not possible, the experiments did report positive values, therefore, confirming the presence of cationic charges on those vesicles). 

### 3.5. Small X-ray Scattering (SAXS)

**Figure 7:**
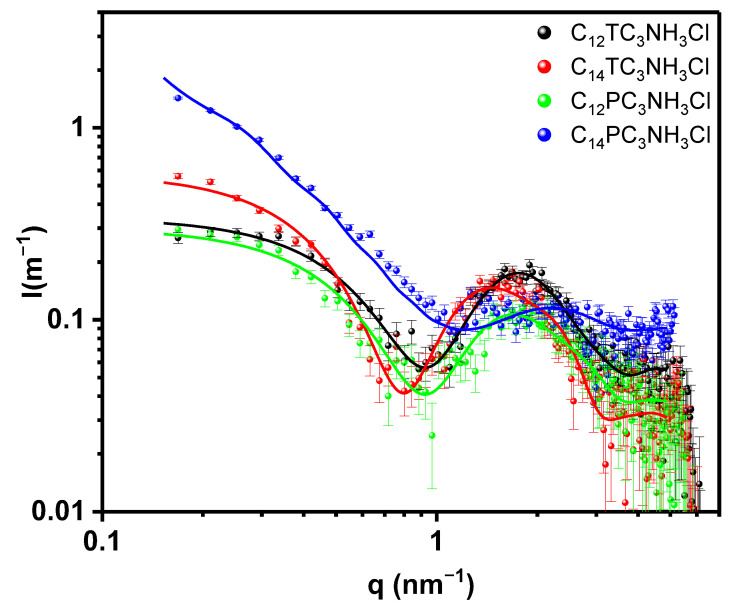
SAXS Scattered intensity as a function of scattering vector modulus q for the different surfactants tested, black squares C_12_TC_3_NH_3_Cl, red circles C_14_TC_3_NH_3_Cl, green triangles C_12_PC_3_NH_3_Cl, and blue down triangles C_14_PC_3_NH_3_Cl. The lines correspond to the best fit of core-shell cylindrical models with the parameters shown in Table 2.

The formulations of cationic vesicles were further studied using SAXS. First, we consider the self-aggregation of the surfactant molecules by themselves at the concentration of 10 mM. The corresponding scattered intensity as a function of the scattering vector modulus is shown in Figure 7. In the same figure, we show the best fits using a core-shell cylindrical model as lines [23]. Several other models have been tried, such as core-shell spheres, but those other models systematically produced an exceedingly large estimate for the polar headgroups region, which could not be reconciled with observed absolute intensities. In order to reduce the fitting parameters, we have fixed the hydrophobic electronic density to that derived from Tanford’s volumes [54]; therefore, the fitted parameters correspond to the core radius, shell thickness, cylinder length, and polar head electron density. We did not observe any interference effect, which is reasonable for such low concentrations. The parameters of the fit can be observed in Table 2. In light of the reduced chi-squared values and of the plot of the data and model, the fits can be considered satisfactory. Hydrophobic radii are within expectations for a -(CH_2_)_10_CH_3_ or a -(CH_2_)_12_CH_3_ alkyl chain (maximum extended length of 1.4 or 1.7 nm, respectively [54]) with experimental radii within 0.6–0.8 of the maximum length. The cylinder lengths are small except for C_14_PC_3_NH_3_Cl, which is about 60 nm long; this agrees with the macroscopic observation of increased viscosity of this sample. Also, the hydration of this surfactant headgroup is significantly smaller than that of the other three samples. Increased viscosity was observed during titration of C_12_PC_3_NH_3_Cl with NaOH, in line with the tendency of those surfactants to form elongated structures. The area per surfactant molecule is reasonable for single-chain surfactants. The electron density found with the fit and that obtained volumetrically using the calculated number of molecules per surfactant headgroup fairly agree within 10–20 e/nm^3^.

**Figure 8:**
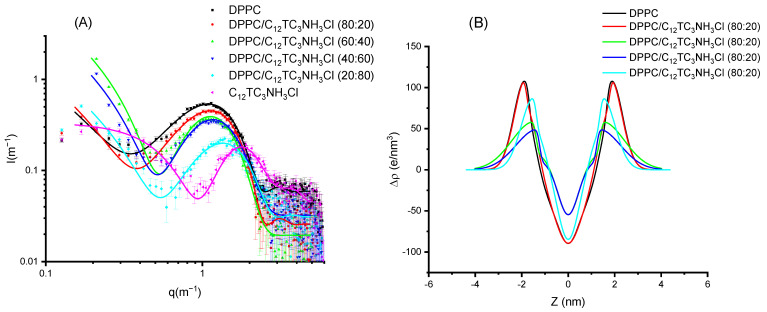
(**A**) Scattered intensity patterns as a function of scattering vector modulus for DPPC and C_12_TC_3_NH_3_Cl and their mixtures; the curves correspond to the best fit of Gaussian bilayers or core-shell models. (**B**) The corresponding electron density profiles of the bilayer models corresponding to the best fits of (**A**).

To further investigate if these surfactants can be incorporated into cationic vesicles, we mixed these compounds with DPPC. The samples were prepared to induce the formation of vesicles. DPPC produces a scattering pattern typical of vesicles (see Figure 8) with a wide bump centered around a *q* value of 0.1 Å^−1^. This scattering pattern is due to the electronic distribution present in phospholipid bilayers with a low electronic density region at the center of the bilayer (corresponding to the methyl and methylene groups of the hydrophobic chains) and two high electronic density regions corresponding to the location of the phosphatidyl-choline groups [22]. This can be represented by using a Gaussian description of the bilayer, and the X-ray spectra correspond to the Fourier Transform of this electronic distribution. The addition of surfactant produces a displacement of the bump to higher *q* values (i.e., smaller distances). In Figure 8A, the experimental results are shown for DPPC and their mixtures with C_12_TC_3_NH_3_Cl, together with the best fit of these data. The surfactant alone is also shown for comparison; however, this could not be sensibly fit to a bilayer model. In Figure 8B, the corresponding electron density profiles are also shown. The parameters of the fits for those Gaussian bilayers are given in Table 3 and Table 4.

**Figure 9:**
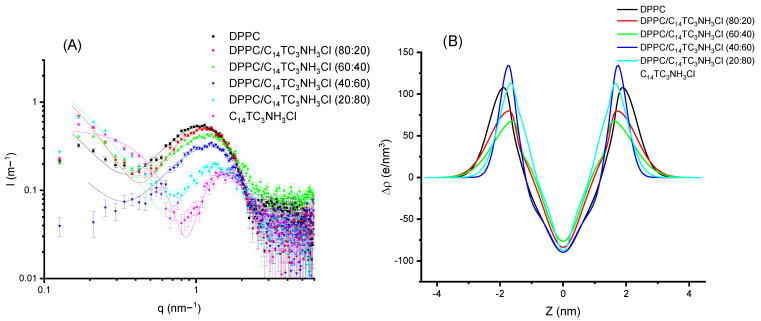
(**A**) Scattered intensity patterns as a function of scattering vector modulus for DPPC and C_14_TC_3_NH_3_Cl and their mixtures; the curves correspond to the best fit of Gaussian bilayers or core-shell models. (**B**) The corresponding electron density profiles of the bilayer models corresponding to the best fits of (**A**).

Comparing Figure 8 and Figure 9, few differences can be appreciated. When looking in detail, the band for C_12_TC_3_NH_3_Cl appears at a slightly smaller q than that of C_14_TC_3_NH_3_Cl. Some more different are the electronic density profiles. 

From these results, we can conclude that the surfactants incorporate into the bilayers without producing dramatic changes in the structures of the bilayer up to high surfactant contents. All of the samples were unilamellar within the detection sensitivity, which can be estimated to be about 10%. Very similar results were found for C_12_PC3NH_3_Cl and C_14_PNH_3_Cl. Both the graphs and parameters are shown in the Electronic Supporting Information File.

### 3.6. Antimicrobial Activity

The antibacterial activity was tested against eight representative bacteria strains (*Bacillus subtillis*, *Staphylococcus epidermidis*, *Staphylococcus aureus*, *Listeria monocytogenes*, *Enterococcus faecalis*, *Escherichia coli*, *Acinetobacter baumannii*, and *Klebsiella aerogenes*). The concentration tested for all surfactants ranged from 500 to 2 µM. The obtained MIC values (concentration required to completely inhibit the growth of microorganisms), as well as the MBC values (concentration of surfactants required to kill microorganisms), are summarized in Table 5 and Table 6. The MIC values of benzalkonium chloride (BAC), the most widely used quaternary ammonium antiseptic, are also given in Table 5 as a reference.

In general, these surfactants exhibit good activity against Gram-positive bacteria and moderate activity against Gram-negative. Usually, the mode of action of cationic surfactants involves the electrostatic and hydrophobic interaction with bacterial cell membranes. This mechanism explains the broad-spectrum activity of these compounds as well as the tolerance of Gram-negative bacteria to these antimicrobials. While Gram-positive bacteria possess a single phospholipid cell membrane and a thicker cell wall composed of peptidoglycan, Gram-negative bacteria are encapsulated by two cell membranes and a rather thin layer of peptidoglycan [55], and this makes the interaction of some antimicrobials with this target difficult.

As expected, the antimicrobial activity of these surfactants depends on the alkyl chain length, which is associated with their hydrophobic character. In general, the shortest homolog for the tryptophan series, the C_8_ derivative, exhibited scarce activity against the tested microorganisms with MIC values ≥ 500 μM, while the C_10_ homologs showed moderated activity with MIC values in the range of 62–500 μM. Phenylalanine and tryptophan derivatives containing C_12_ and C_14_ alkyl chains were the most effective. These compounds exhibited activity against all microorganisms tested, including the Gram-negative bacteria. This behavior has already been described for numerous surfactant series. Some biological properties, such as antimicrobial activity, exhibit a nonlinear dependence on the hydrophobic alkyl chain length. This phenomenon is known as the cutoff effect, and usually, for most of the reported surfactant series, the C_12_-C_14_ homologs display the best effectiveness [15]. 

From the MIC values obtained, it can be stated that the amino acid used in the polar head does not have a significant effect on antibacterial activity. This can be ascribed to the similar CMC and, consequently, hydrophobic–hydrophilic character obtained for homologs with the same alkyl chain. As we discussed above, the hydrophobic character of surfactants is one of the structural features that seriously affects their antimicrobial activity. Recently, our group has reported the antimicrobial properties of cationic surfactants containing two amino acids on the polar heads, namely, phenylalanine-arginine and tryptophan-arginine [36]. The best activity was also found for the C_12_ homologs, and their activity is similar to that shown by C_12_PC_3_NH_3_Cl and C_12_TC_3_NH_3_Cl, the most effective surfactants of the series described in the present work. 

Jondan et al., developed phenylalanine derivatives with different head groups in which the alkyl chain is linked to the carboxylic group of the amino acid through an ester bond [56]. It is expected that, given their chemical structure, these surfactants have good biodegradation levels; however, the ester bond present in their structures can compromise their chemical stability. The antimicrobial activity reported for the homologs containing the positive charge in the protonated amine group of the phenylalanine is clearly lower than that displayed by the C_n_PC_3_NH_3_Cl and C_n_TC_3_NH_3_Cl. However, the antimicrobial activity increases significantly when the α-amine group of these phenylalanine derivatives is tri-ethylated and the positive charge does not depend on the pH [57].

The MIC values reported for some tryptophan alkyl amide compounds (≤1 for Gram-positive and ≤6 for Gram-negative bacteria) [58] and dipeptide amphiphiles with varying head groups, including the phenylalanine and tryptophan (≤0.5 for Gram-positive and ≤10 for Gram-negative) [59], were lower than those obtained for the phenylalanine and tryptophan homologs described in this work. The advantage of the present compounds is that they can be prepared using an environmentally friendly synthetic procedure. The preparation of the cited dipeptide amphiphiles requires a multistep synthesis using protecting amino acids and activating agents.

Different results have been reported regarding the effect of the aromatic group on antibacterial activity. It seems that the presence of aromatic rings enhances the antimicrobial effectiveness. For example, benzalkonium bromide, a compound with a benzyl group on the polar head, showed higher activity than its counterpart, dodecyl trimethyl ammonium [60,61], and cationic surfactants containing two aromatic rings on their polar head (tryptophan-proline) exhibited very low MIC values against Gram-positive bacteria (MIC 0.1 µg/mL) compared to phenylalanine-proline derivatives with only one aromatic ring (MIC 1 µg/mL) [59].

The MBC of these cationic surfactants, the concentration at which >99.9% of bacteria are killed, is also displayed in Table 5 and Table 6. The obtained MBC/MIC ratio ranged from 1 to 2, indicating that these antimicrobials presented a potent bactericidal activity against these microorganisms. This means that these compounds not only inhibit the growth of bacteria but also kill these microbes. The antimicrobial activity has also been determined for the vesicle formulations. 

Table 7 shows the MICs values corresponding to the formulations containing DPPC/C_12_TC_3_NH_3_Cl. The MICs of this table correspond to the concentration of the cationic surfactant in the formulation. The antimicrobial activity of these systems depends on the ratio of DPPC/surfactant. The formulation with 80% of C_12_TC_3_NH_3_Cl exhibited similar antimicrobial activity to the pure surfactants. This means that these vesicles can act as carriers and antimicrobial agents simultaneously. The incorporation of higher percentages of DPPC in the formulations results in an important increase in the MIC values. In fact, formulations containing only 20% of this cationic surfactant did not show activity against any of the tested bacteria. This behavior was also observed for diacyl-glycerol arginine-based surfactants; the pure cationic surfactant exhibited good antimicrobial activity, while its vesicles had very low effectivity against bacteria [30]. These results indicate that the additive, and consequently the effect of the additive on the physicochemical properties, plays an important role in the antibacterial activity of these systems. It has been described that the antimicrobial activity of surfactants shows dependence on aggregate structure, where the aggregates with small size show better antimicrobial efficiency than that of large size. Moreover, the micelles act as monomer reservoirs, and they continuously release surfactant monomers, which effectively interact with bacterial membranes [44]. The SAXS studies indicate that the 20/80 formulation contains vesicles, and the microscope observations suggest that these aggregates have a large size. However, considering its visual aspect (it is totally transparent) as well as the high content of the cationic surfactants, it would be expected that these systems contain a mixture of vesicles and micelles, and because of that, they exhibit good antibacterial activity. The presence of micelles probably decreases drastically as the percentage of DPPC increases; in fact, these formulations show the typical opalescence of liposomal systems. These changes in the aggregate size and stability could be related to the low activity shown by formulations containing more than 40% of DPPC. These results agree with those published by Carmona–Ribeiro et al. These authors found that small DODAB bilayer fragments (D_h_ = 79 nm) penetrated more deeply into the cell surfaces and, consequently, had lower MIC values than DODAB large vesicles (D_h_ = 500–800 nm) [62]. Cationic charge density is another physicochemical parameter that affects antimicrobial activity. Increasing the number of cationic charges in the hydrophilic group is a common strategy to improve antimicrobial activity [63]. It was also found that the antimicrobial activity of catanionic vesicles depended on the percentage of cationic surfactants [64]. In this regard, it is expected that vesicles containing a low percentage of cationic surfactant have lower cationic charge density; this property will prevent the electrostatic interaction of these aggregates with the negatively charged bacterial walls. 

The physicochemical properties of the other vesicular systems DPPC/C_14_TC_3_NH_3_Cl, DPPC/C_12_PC_3_NH_3_Cl, and DPPC/C_12_PC_3_NH_3_Cl are not very different from those shown by the DPPC/C_12_TC_3_NH_3_Cl, however, unexpectedly, these formulations did not exhibit effectivity against any of the tested bacteria. This behavior can be ascribed to the different affinity between the surfactant molecules with DPCC during vesicle formation (see Section 3.8). 

From the obtained results, it can be stated that with these two amino acid-based surfactant series, it is possible to generate cationic vesicles with different antimicrobial activities. On the one hand, vesicles with antimicrobial activity can be used as vehicles and drugs simultaneously or can be used to load another drug in order to have antimicrobial systems with two therapeutic applications. On the other hand, systems without antimicrobial activity can be used to load drugs for biomedical applications that do not require bacteriostatic activity. 

### 3.7. Hemolytic Activity

Hemolysis is one of the most widely used cell membrane systems to determine surfactant biocompatibility, which is especially significant in their biomedical applications. The HC_50_ values of the C_n_PC_3_NH_3_Cl and C_n_TC_3_NH_3_Cl (concentration that induces 50% hemolysis of the erythrocytes) was calculated from plots of % hemolysis as a function of surfactant concentration (see Table S3 and Figure S45). For the tryptophan derivatives, it was found that the hemolytic activity increased considerably when the alkyl chain went from 10 to 12 carbon atoms. After that, a slight growth in this activity was found for the C_14_ derivative. The influence of the hydrophobic chain length on cytotoxicity is still not well understood. Given that erythrocytes lack internal organelles, the only way for cationic surfactants to interact with them is via the cell membrane. The most accepted hypothesis is that surfactants interact with the erythrocyte membranes through electrostatic and hydrophobic interactions. The hydrophobic moiety of these molecules modulates the penetration of the surfactant hydrophobic part that is responsible for the membrane disturbance due to the curvature changes [65]. This theory agrees with the results obtained for the tryptophan derivatives, and the literature describes several surfactant analogs [66], including aromatic-based amino acids [57], in which hemolysis increases with the elongation of the hydrophobic chain. However, there are also some works describing surfactant homologs in which hemolysis decreases as the alkyl chain increases [58].

The increase in the hydrophobic character of the phenylalanine homologs from C_10_ to C_12_ causes an important intensification in their ability to interact with erythrocytes. However, a large decrease in the hemolytic activity was observed for the C_14_ derivative. This behavior could be attributed to the aggregates’ shape and size. Aqueous solutions containing the C_14_ homolog are very viscous; this viscosity suggests the presence of large micelles, probably threat-like aggregates of large size. Similar behavior was observed for arginine-based gemini surfactants [67]. In this regard, Vieira and Carmona–Ribeiro also observed that quaternary ammonium single-chain surfactant solutions containing micelles were more hemolytic than those of large vesicles formed by their double-chain quaternary homolog [62].

The biomedical applications of cationic surfactants depend on their ability to selectively interact with bacterial membranes without toxic effects on human cells. The phenylalanine C_10_ homolog did not exhibit selectivity against bacteria, while the tryptophan C_10_ derivatives showed moderate selectivity against the Gram-positive bacteria. The two C_12_ derivatives exhibited antimicrobial activity against practically all bacteria tested at a concentration below that provoking hemolysis. These surfactants showed a good therapeutic window with HC_50_/MIC ratios ≥ 2 for all Gram-positive bacteria and for *E. coli* and K. aerogenes. It is noteworthy that the C_14_ phenylalanine showed very low hemolytic character; at the maximum concentration tested (200 μM), only about 30% of hemolysis was obtained. This means that this surfactant shows excellent selectivity against Gram-positive bacteria and *A. baumani*.

### 3.8. Molecular Docking

The major component of the bacterial cell wall is a cross-linked glycopeptide polymer called peptidoglycan. This polymer surrounds the cytoplasmic membrane of bacteria and functions as an exoskeleton, maintaining cell shape and stabilizing the membrane against fluctuations in osmotic pressure. A functioning peptidoglycan pathway is required for bacterial cell growth and division, and compounds that inhibit peptidoglycan biosynthesis have antibiotic activity [68].

Molecular docking studies were carried out to understand the observed antimicrobial activities of the prepared surfactants and to shed light on the binding modes between docked ligands and enzyme targets [69,70,71].

**Figure 10:**
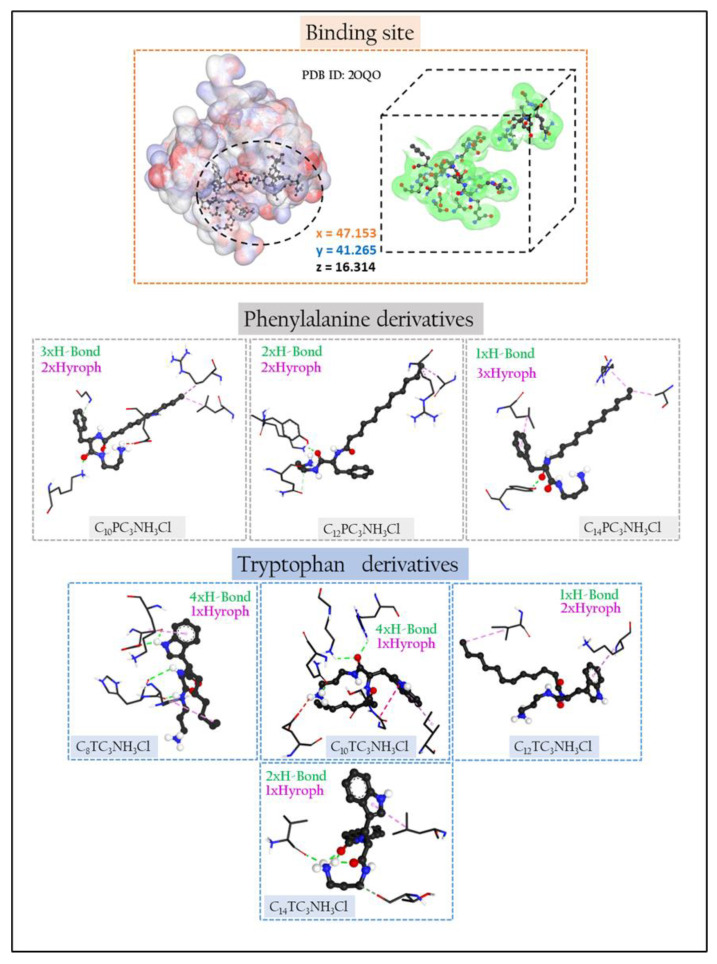
Three-dimensional (3D) closest interactions between active site residues of peptidoglycan glycosyltransferase (PDB ID:2OQO) with phenylalanine and tryptophan surfactants derivatives.

The likeliest docked positions of surfactant ligands with the best binding affinity for ligands complexes in the active site of the targeted receptor are shown in Figure 10. For Cn benzalkonium derivatives, their mode of interaction is shown in Figure S46. The docking results of the synthesized compounds with the enzyme’s targets have given good information about the nature of the binding mode.

All surfactants showed a good affinity to receptor pocket (Table 8 and Table 9), with free energy binding values between −6 and −7.9 kcal/mol, thus representing a good affinity with respect to Cn benzalkonium derivatives (between −5.8 and −7.2 kcal/mol, see Table S4). The better score obtained for the amino acid derivatives as compared with benzalkonium derivatives is probably due to the presence of hydrogen bonding in the former and their absence in the latter.

The phenylalanine and tryptophan-based surfactants show three types of interactions: electrostatic, hydrogen bonding, and hydrophobic. The functional groups of the molecules, carbonyl, secondary amine NH_2_, hydrophobic parts, and aromatic rings participate in the mode of interaction against different residues in the receptor pocket.

The elongation of the hydrophobic alkyl chain does not affect the number of hydrophobic interactions. For the phenylalanine derivatives, the hydrophobic interactions move from 2 to 3 when the alkyl chain increases from C_12_ to C_14_. Similar behavior has been observed for the C_10_ and C_12_ tryptophan derivatives, while the transition from C_12_TC_3_NH_3_Cl to C_14_TC_3_NH_3_Cl leads to a decrease in the number of hydrophobic bonds from 2 to 1.

All inhibitors show different numbers of interactions, except the C_10_TC_3_NH_3_Cl, C_14_TC_3_NH_3_Cl, and C_10_PC_3_NH_3_Cl, which present attractive charge interactions in their mode of action. It is not easy to justify a single effect or a combination of effects that can fully explain the differences in reactivity of the ligand in the protein pocket. The attractive–charge interaction distances observed in the mode of action of some ligands can be attributed to the existence of intramolecular interaction Charge-Dipole type between the N^+^ positive charge on the atom and the lone pair of the oxygen atoms attached to the carbonyl’s functions. This can also be explained by the degree of steric hindrance which exists at the level of the polar heads of the surfactants, which will prevent the contribution of N^+^ in the electrostatic interaction with the amino acid residues.

### 3.9. DFT Calculation

**Figure 11:**
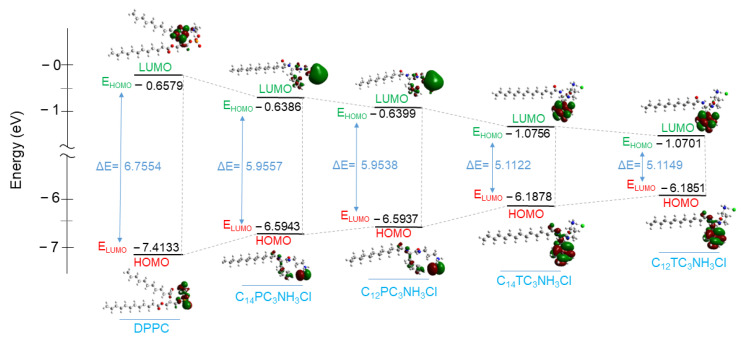
Molecular Orbitals diagram showing HOMO and LUMO levels and the HOMO − LUMO gap of DPPC, C_14_PC_3_NH_3_Cl, C_12_PC_3_NH_3_Cl, C_14_TC_3_NH3Cl, and C_12_TC_3_NH_3_Cl.

Several electronic parameters in terms of chemical descriptors were calculated based on the geometrical optimization of the new surfactants molecules: energy of the highest occupied molecular orbital (E_HOMO_), the energy of the low unoccupied molecular orbital (E_LUMO_), energy gap (ΔE), hardness (ɳ), and electronegativity (χ). These parameters can help to correlate the electronic distribution of the DPPC/Surfactants vesicles and their antibacterial activity. The parameter values are shown in Table 10, and the frontier molecule orbital density is presented in Figure 11.

All these parameters were assessed to elucidate the DPPC interaction with the C_12_ and C_14_ derivatives. The E_HOMO_ (higher energy) is often associated with the ability of the molecule to donate the electron, and the E_LUMO_ (lowest energy) indicates the higher probability that the molecule would accept electrons [72]. It can be observed that the E_HOMO_ of these surfactants is less negative (−6.1851eV to −6.5943 eV) than that of the DPPC (−7.4133 eV). This indicates that surfactant molecules are more likely to give electron density than DPPC molecules. Then, it is expected that when the pairs of DPPC/Surfactants interact, the tendency of electronic charge transfer will be performed easily from the surfactant’s molecules to the DPPC.

The gap energy ΔE = E_LUMO_ − E_HOMO_ reflects the polarizability and the reactivity of a molecule [73,74]. The ΔE obtained for all surfactants is lower than that obtained for the DPPC, reflecting more affinity of the negative charge from surfactants towards the DPPC molecule. The other parameters, such as electronegativity (χ) and hardness (ɳ), also proved that the electronic charge transfer is from the surfactant molecules to the DPPC. The electronegativity (χ) is an important indicator that can be used in structural and reactivity studies. This property represents the electron-pulling power of molecules; therefore, molecules with low electronegativity values can give electrons easily [75]. The investigated surfactants have lower electronegativity values (3.61 eV to 3.62 eV) than DPPC (4.03 eV), which also indicates that the DPPC/surfactant interaction leads to a transfer of electronic density from cationic surfactants to the DPPC.

The Hard–Soft–Acid–Base theory (HSAB) classifies reactive species as relatively “hard” or “soft” based on polarizability, i.e., the ease with which electronic density can be shifted or delocalized [76]; hard molecules are less reactive than soft molecules because they require more energy for molecular excitation [77]. DPPC shows higher chemical hardness (ɳ) than all cationic surfactants (Table 10), which also evidences that the electron density at the HOMO molecular orbital levels of the surfactants is soft. This parameter also indicates the flexibility of electronic density from surfactants to the DPPC molecules.

From the calculated chemical descriptors, it can be assumed that when DPPC interacts with those surfactants, DPPC molecules play the role of electron density acceptor while surfactant molecules act as donors.

The loss of the biological activity of these liposomes can be attributed to the strong molecular affinity between DPPC and surfactants. This interaction affinity between DPPC and surfactant molecules can be determined by calculating interaction energies (noted E_int_) according to D.C. Young [78] by using Equation (4).

(4) $$E_{\mathrm{int}}={\;E}_{\mathrm{DPPC}\_\mathrm{Surfactant}}-\left(E_{\mathrm{DPPC}}+E_{\mathrm{Surfactant}}\right)$$

where E_DPPC_ is the total energy of the DPPC molecule, E_Surfactant_ is the total energy of the surfactants compounds, and E_DPPC_Surfactant_ represents the total energy of the new complex system formed between the surfactants and the DPPC.

**Figure 12:**
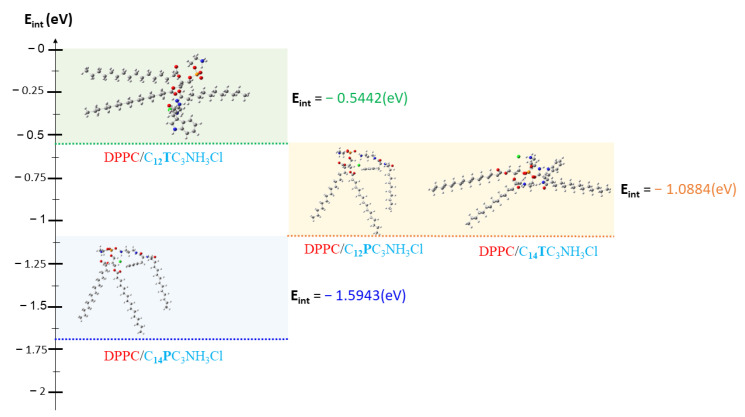
Interaction Energies between the surfactants and DPPC.

All complexes formed between DPPC and surfactants present negative energies of interaction (Figure 12, Table 10), indicating a good affinity between DPPC and surfactants in vesicle formation. However, it should be noted that the energy calculated for DPPC/C_12_TC_3_NH_3_Cl is higher than that of the other three complexes, which reflects a lower affinity between DPPC and C_12_TC_3_NH_3_Cl. Due to the low affinity between C_12_TC_3_NH_3_Cl and DPPC, the cationic surfactants in these formulations will be more available to interact with the bacterial walls and, consequently, maintain some antibacterial activity. However, for the C_12_PC_3_NH_3_Cl, C_14_PC_3_NH_3_Cl, and C_14_TC_3_NH_3_Cl, the strong affinity between the surfactant molecules with DPCC in the vesicle will reduce the availability of surfactant molecules to be active against bacteria. Shao et al. found that the strong interactions between phospholipids vesicles and ionic-charged chitosan/alginate multilayers give rise to perfect fluid lipid bilayers on polyelectrolytes multilayers. Coarse-grained molecular dynamics simulations suggested that the formation of these fluid bilayers follows the parachute model [79]. A similar situation may occur when the mixed vesicles interact with bacterial membranes.

## 4. Conclusions

Cationic amino-acid based-surfactants have been successfully prepared using tryptophan and phenylalanine. The synthetic approach used to prepare these compounds fits some of the green chemistry requirements: utilization of renewable starting materials and a synthetic procedure consisting of two simple chemical reactions that do not require the use of an organic solvent or protecting amino acids.

Both headgroups can be considered weak acids, where apparent pK_a_ decreases as the hydrophobic chain length increases, with phenylalanine being consistently more acidic than tryptophan. The CMC of the surfactant was determined primarily by the hydrophobic/hydrophilic balance. It was found that the presence of the indole group in the structure of tryptophan surfactants increases the hydrophilic character and, consequently, the CMC of the C_n_TC_3_NH_3_Cl homologs. The micelles of both families of surfactants based on dodecyl and tetradecyl hydrophobic chains can be fitted to cylindrical models, with C_14_PC_3_NH_3_Cl producing the most elongated micelles, agreeing with the macroscopically observed viscosity of their solutions.

SAXS investigations show the formation of unilamellar vesicles for all DPPC/surfactant ratios; moreover, only the bilayer thickness of vesicles with higher surfactant contents was significantly affected. Surfactants with C_12_-C_14_ alkyl chains exhibited good antimicrobial activity against Gram-positive and Gram-negative bacteria, and the type of amino acid did not affect their antimicrobial activity. Vesicles containing 60 and 80% of C_12_TC_3_NH_3_Cl exhibited good antimicrobial activity; the effectiveness against these microorganisms decreased drastically when the percentage of DPPC increased. However, vesicles containing phenylalanine derivatives and the C14 tryptophan homolog did not show activity at the tested concentrations. DFT simulation suggests that the lack of activity observed for these vesicles could be ascribed to the high affinity between DPPC and these surfactants.

Molecular docking results obtained using the enzyme peptidoglycan glycosyltransferase suggest the existence of three types of interactions that could be the basis of some of the antimicrobial mechanisms.

## Figures and Tables

**Table 1:** Apparent pKa values and micellization parameters of C_n_TC_3_NH_3_Cl and C_n_PC_3_NH_3_Cl. Values are given with 95% confidence intervals.

Compound	pK_a_	CMC(κ) Conductivity mM	CMC(I_1_/I_3_) Fluorescence mM	β
C_8_TC_3_NH_3_Cl	9.89 ^2^	-	5.0 ± 1.0	-
C_10_TC_3_NH_3_Cl	9.35 ^2^	7.3 ± 0.1	1.3 ± 0.1	0.26 ± 0.01
C_12_TC_3_NH_3_Cl	7.95 ^1^ 7.66 ^2^	1.72 ± 0.05	0.67 ± 0.15	0.26 ± 0.01
C_14_TC3NH_3_Cl	6.96 ^1^ 6.89 ^2^	0.55 ± 0.05	0.46 ± 0.04	0.48 ± 0.03
C_10_PC3NH_3_Cl	9.06 ^2^	4.1 ± 0.4	3.0 ± 0.1	0.11 ± 0.01
C_12_PC3NH_3_Cl	7.70 ^1^ 7.53 ^2^	1.76 ± 0.06	0.4 ± 0.2	0.26 ± 0.02
C_14_PC3NH_3_Cl	6.55 ^1^ 6.70 ^2^	0.34 ± 0.02	0.20 ± 0.05	0.28 ± 0.01

^1^ pK_a_ obtained in the titration with NaOH. ^2^ pK_a_ obtained in the back titration with HCl.

**Table 2:** Parameters of the models used to fit the surfactants SAXS.

	C_12_TC_3_NH_3_Cl	C_14_TC_3_NH_3_Cl	C_12_PC_3_NH_3_Cl	C_14_PC_3_NH_3_Cl
$\chi_{red}^{2}$	1.07	1.85	1.15	3.5
φ	0.00187	0.0022	0.00187	0.0022
R_h_ (nm)	0.80 ± 0.05	0.97 ± 0.07	0.92 ± 0.05	0.65 ± 0.1
R_c_ (nm)	1.04 ± 0.05	1.12 ± 0.07	0.96 ± 0.05	1.15 ± 0.1
L_c_ (nm)	5.9 ± 0.5	8.5 ± 0.8	6.8 ± 0.5	64 ± 10
ρ_h_ (e/nm^3^)	371 ± 10	369 ± 10	363 ± 10	395 ± 15
ρ_c_ (e/nm^3^)	274	277	274	277
N_Agg_	61 ± 5	88 ± 0.05	63 ± 0.05	690 ± 0.05
N H_2_O	18 ± 3	28 ± 0.05	29 ± 0.05	7 ± 0.05
A_m_ (nm)	0.63 ± 0.05	0.68 ± 0.05	0.65 ± 0.05	0.67 ± 0.05

$\chi_{red}^{2}$ reduced chi-square, φ volume fraction of hydrocarbon chains, R_h_ hydrophilic thickness, R_c_ hydrocarbon core radius, L_c_ cylinder length, ρ_h_ hydrophilic electron density, ρ_c_ hydrocarbon electron density, N_Agg_ number of aggregation, N H_2_O number of water molecules per headgroup, and A_m_ area per molecule at the hydrophilic/hydrophobic interface.

**Table 3:** Fitting parameters of Gaussian bilayers for DPPC/C_12_TC_3_NH_3_Cl mixtures.

	DPPC/C_12_TC_3_NH_3_Cl				
	DPPC	80%/20%	60%/40%	40%/60%	20%/80%
$\chi_{red}^{2}$	1.19	0.72	4.77	2.90	1.35
σ_h_ (nm)	0.47 ± 0.05	0.50 ± 0.05	0.95 ± 0.1	0.93 ± 0.08	0.55 ± 0.05
Δρ_h_ (e/nm^3^)	107 ± 10	111 ± 10	58 ± 15	48 ± 15	87 ± 10
Z_h_ (nm)	1.87 ± 0.05	1.80 ± 0.05	1.47 ± 0.05	1.33 ± 0.05	1.47 ± 0.05
σ_c_ (nm)	0.43 ± 0.10	0.48 ± 0.10	0.43 ± 0.15	0.41 ± 0.15	0.38 ± 0.10

**Table 4:** Fitting parameters of Gaussian bilayers for DPPC/C_14_TC_3_NH_3_Cl mixtures.

	DPPC/C_14_TC_3_NH_3_Cl				
	DPPC	80%/20%	60%/40%	40%/60%	20%/80%
$\chi_{red}^{2}$	1.19	1.83	3.08	1.73	4.22
σ_h_ (nm)	0.47 ± 0.05	0.63 ± 0.05	0.69 ± 0.08	0.32 ± 0.05	0.47 ± 0.08
Δρ_h_ (e/nm^3^)	107 ± 10	79 ± 10	69 ± 13	137 ± 10	119 ± 15
Z_h_ (nm)	1.87 ± 0.05	1.69 ± 0.05	1.50 ± 0.05	1.70 ± 0.05	1.54 ± 0.05
σ_c_ (nm)	0.43 ± 0.10	0.43 ± 0.10	0.48 ± 0.13	0.40 ± 0.10	0.25 ± 0.07

**Table 5:** MIC/MBC (µM) values of studied N^α^-acyl-tryptophan salts.

Bacteria Strain	C_8_TC_3_NH_3_Cl		C_10_TC_3_NH_3_Cl		C_12_TC_3_NH_3_Cl		C_14_TC_3_NH_3_Cl		BAC
	MIC	MBC	MIC	MBC	MIC	MBC	MIC	MBC	MIC
Gram-negative									
*A. baumannii*	500	>500	500	500	250	250	125	125	62
*E. coli*	500	>500	125	125	31	62	125	500	62
*K. aerogenes*	>500	>500	500	500	125	125	250	250	62
Gram-positive									
*S. epidermidis*	250	500	62	250	16	31	8	31	16
*E. faecalis*	500	>500	62	125	16	62	16	31	8
*S. aureus*	250	500	62	62	8	8	4	16	16
*L. monocytogenes*	500	>500	250	250	62	62	31	31	62
*B. subtilis*	250	500	125	125	15	31	8	31	16

**Table 6:** MIC/MBC (µM) values of studied N^α^-acyl-phenylalanine salts.

Bacteria Strain	C_10_PC_3_NH_3_ Cl		C_12_PC_3_NH_3_ Cl		C_14_PC_3_NH_3_ Cl	
	MIC	MBC	MIC	MBC	MIC	MBC
Gram-negative						
*A. baumannii*	>500	>500	125	125	62.5	125
*E. coli*	250	250	31	31	500	500
*K. aerogenes*	500	500	31	31	500	500
Gram-positive						
*S. epidermidis*	125	125	31	31	31	125
*E. faecalis*	250	500	16	31	31	62
*S. aureus*	250	250	31	125	62	125
*L. monocytogenes*	500	500	31	31	31	62
*B. subtilis*	250	500	31	31	62	125

**Table 7:** MIC/MBC (µM) values of DPPC/C_12_TC_3_NH_3_Cl formulations.

Bacteria Strain	DPPC/C_12_TC_3_NH_3_Cl							
	80%/20%		60%/40%		40%/60%		20%/80%	
	MIC	MBC	MIC	MBC	MIC	MBC	MIC	MBC
Gram-negative								
*A. baumannii*	>570	>570	>570	>570	>570	>570	380	380
*E. coli*	>570	>570	570	>570	380	>570	47	47
*K. aerogenes*	>570	>570	570	>570	380	>570	47	47
Gram-positive								
*S. epidermidis*	>570	>570	570	>570	380	>570	23	23
*E. faecalis*	>570	>570	570	>570	380	>570	23	23
*S. aureus*	>570	>570	570	>570	380	>570	23	23
*L. monocytogenes*	>570	>570	-	>570	-	>570	190	380
*B. subtilis*	>570	>570	570	>570	380	>570	23	23

**Table 8:** Results of the interaction details and docking score in (kcal/mol) of the phenylalanine ligands derivatives against the peptidoglycan glycosyltransferase (PDB ID:2OQO).

Inhibitors	Score Kcal/mol	Ligand	Receptor	Interactions		Distance (Å)
				Categories	Types	
C_10_PC_3_NH_3_Cl	−6.7	H(N^+^H_3_)	GLU83	Hydrogen Bond; Electrostatic	Salt Bridge; Attractive Charge	2.34
		O(C=O)	LYS124	Hydrogen Bond	Conventional Hydrogen Bond	1.99
		H(Ring)	GLY115	Hydrogen Bond	Pi-Donor Hydrogen Bond	3.13
		C(CH3)	LEU221	Hydrophobic	Alkyl	3.97
		C(CH3)	ARG225	Hydrophobic	Alkyl	4.19
C_12_PC_3_NH_3_Cl	−7.9	O(C=O)	TYR206	Hydrogen Bond	Conventional Hydrogen Bond	2.52
		H(CH2)	GLN217	Hydrogen Bond	Carbon Hydrogen Bond	3.31
		C(CH3)	ALA97	Hydrophobic	Alkyl	3.72
		C(CH3)	ARG85	Hydrophobic	Alkyl	4.34
C_14_PC_3_NH_3_Cl	−7.6	O(C=O)	TYR206	Hydrogen Bond	Conventional Hydrogen Bond	2.37
		C(CH3)	ALA97	Hydrophobic	Alkyl	3.62
		Ring	ARG85	Hydrophobic	Alkyl	4.21
		C(CH3)	LEU221	Hydrophobic	Pi-Alkyl	4.87

**Table 9:** Results of the interaction details and docking score in (kcal/mol) of the tryptophan ligand derivatives against the peptidoglycan glycosyltransferase (PDB ID:2OQO).

Inhibitors	Score Kcal/mol	Ligand	Receptor	Interactions		Distance (Å)
				Categories	Types	
C_8_TC_3_NH_3_Cl	−6.7	H(NH indole)	GLU148	Hydrogen Bond	Conventional Hydrogen Bond	2.84
		H(NH indole)	GLU148	Hydrogen Bond	Conventional Hydrogen Bond	1.81
		H(NH)	HIS89	Hydrogen Bond	Conventional Hydrogen Bond	2.17
		H(NH)	HIS90	Hydrogen Bond	Conventional Hydrogen Bond	2.95
		C(CH3)	HIS89	Hydrophobic	Pi-Alkyl	5.17
		Ring	LYS153	Hydrophobic	Pi-Alkyl	5.22
C_10_TC_3_NH_3_Cl	−7	H(N^+^H_3_)	GLU83	Hydrogen Bond; Electrostatic	Salt Bridge; Attractive Charge	3.09
		O(C=O)	LYS124	Hydrogen Bond	Conventional Hydrogen Bond	2.50
		O(C=O)	ARG132	Hydrogen Bond	Conventional Hydrogen Bond	2.22
		H(N^+^H_3_)	GLN121	Hydrogen Bond	Conventional Hydrogen Bond	2.11
		Ring(C_5_)	GLY115	Hydrophobic	Amide-Pi Stacked	4.72
		Ring(C_6_)	ILE98	Hydrophobic	Pi-Alkyl	5.47
C_12_TC_3_NH_3_Cl	−7.3	C(CH_3_)	ILE145	Hydrophobic	Alkyl	5.00
		Ring(C_6_)	LYS153	Hydrophobic	Pi-Alkyl	4.55
C_14_TC_3_NH_3_Cl	−7.8	H(N^+^H_3_)	VAL112	Hydrogen Bond	Conventional Hydrogen Bond	2.21
		H(CH2)	THR82	Hydrogen Bond	Carbon Hydrogen Bond	3.32
		Ring(C_5_)	LEU221	Hydrophobic	Pi-Alkyl	5.22

**Table 10:** Electronic parameters of studied Surfactants and Interaction energy of DPPC_Surfactants complexes obtained by DFT/B3LYP 6-311 g(d) calculations.

	ɳ (eV)	χ (eV)	E_Int_ (eV)
C_14_PC_3_hNH_3_Cl	2.9778	3.6164	−1.5945
C_12_PC_3_NH_3_Cl	2.9769	3.6168	−1.0884
C_14_TC_3_NH_3_Cl	2.5561	3.6317	−1.0884
C_12_TC_3_NH_3_Cl	2.5574	3.6276	−0.5442
DPPC	3.3777	4.0356	

## Data Availability

The data presented in this article are available within this article.

## Supplementary Materials

The following supporting information can be downloaded at: https://www.mdpi.com/article/10.3390/pharmaceutics15071856/s1. Figure S1: ^1^H NMR spectrum of C_8_TC_3_NH_3_C. Figure S2: ^13^C NMR spectrum of C_8_TC_3_NH_3_Cl. Figure S3: ESI-MS spectrum of C_8_TC_3_NH_3_Cl. Figure S4: ^1^H NMR spectrum of C_10_TC_3_NH_3_C. Figure S5: ^13^C NMR spectrum of C_10_TC_3_NH_3_C. Figure S6: ESI-MS spectrum of C_10_TC_3_NH_3_Cl. Figure S7: ^1^H NMR spectrum of C_12_TC_3_NH_3_C. Figure S8: ^1^H NMR spectrum of C_12_TC_3_NH. Figure S9: ESI-MS spectrum of C_12_TC_3_NH_3_Cl. Figure S10: ^1^H NMR spectrum of C_14_TC_3_NH_3_C. Figure S11: ^13^C NMR spectrum of C_12_TC_3_NH. Figure S12: ESI-MS spectrum of C_14_TC_3_NH_3_Cl. Figure S13: ^1^H NMR spectrum of C_10_PC_3_NH_3_C. Figure S14: ^13^C NMR spectrum of C_10_PC_3_NH_3_C. Figure S15: ESI-MS spectrum of C_10_PC_3_NH_3_C. Figure S16: ^1^H NMR spectrum of C_12_PC_3_NH_3_C. Figure S17: ^13^C NMR spectrum of C_12_PC_3_NH_3_C. Figure S18: ESI-MS spectrum of C_12_PC_3_NH_3_C. Figure S19: ^1^H NMR spectrum of C_14_PC_3_NH_3_C. Figure S20: ^13^C NMR spectrum of C_14_PC_3_NH_3_C. Figure S21: ESI-MS spectrum of C_14_PC_3_NH_3_C. Figure S22: NaOH titration and HCl back titration at 298.15 K for C_8_TC_3_NH_3_Cl. Figure S23: NaOH titration and HCl back titration at 298.15 K for C_10_TC_3_NH_3_Cl. Figure S24: NaOH titration and HCl back titration at 298.15 K for C_12_TC_3_NH_3_Cl. Figure S25: NaOH titration and HCl back titration at 298.15 K for C_14_TC_3_NH_3_Cl. Figure S26: NaOH titration and HCl back titration at 298.15 K for C_10_PC_3_NH_3_Cl. Figure S27: NaOH titration and HCl back titration at 298.15 K for C_12_PC_3_NH_3_Cl. Figure S28: NaOH titration and HCl back titration at 298.15 K for C_14_PC_3_NH_3_Cl. Figure S29: Specific conductivity (κ) as a function of C_10_TC_3_NH_3_Cl concentration. Figure S30: Specific conductivity (κ) as a function of C_12_TC_3_NH_3_Cl concentration. Figure S31: Specific conductivity (κ) as a function of C_14_TC_3_NH_3_Cl concentration. Figure S32: Specific conductivity (κ) as a function of C_10_PC_3_NH_3_Cl concentration. Figure S33: Specific conductivity (κ) as a function of C_12_PC_3_NH_3_Cl concentration. Figure S34: Specific conductivity (κ) as a function of C_14_PC_3_NH_3_Cl concentration. Figure S35: Variation of I_1_/I_3_ ratio of pyrene fluorescence as a function of C_8_TC_3_NH_3_Cl concentration. Figure S36: Variation of I_1_/I_3_ ratio of pyrene fluorescence as a function of C_10_TC_3_NH_3_Cl concentration. Figure S37: Variation of I_1_/I_3_ ratio of pyrene fluorescence as a function of C_12_TC_3_NH_3_Cl concentration. Figure S38: Variation of I_1_/I_3_ ratio of pyrene fluorescence as a function of C_14_TC_3_NH_3_Cl concentration. Figure S39: Variation of I_1_/I_3_ ratio of pyrene fluorescence as a function of C_14_PC_3_NH_3_Cl concentration. Figure S40: Variation of I_1_/I_3_ ratio of pyrene fluorescence as a function of C_12_PC_3_NH_3_Cl concentration. Figure S41: Variation of I_1_/I_3_ ratio of pyrene fluorescence as a function of C_14_PC_3_NH_3_Cl concentration. Figure S42: (A) Scattered intensity patterns as a function of scattering vector modulus for DPPC and C_12_PC_3_NH_3_Cl and their mixtures; the curves correspond to the best fit of Gaussian bilayers or core-shell models. B) The corresponding electron density profiles of the bilayer models corresponding to the best fits of (A). Figure S43: (A) Scattered intensity patterns as a function of scattering vector modulus for DPPC and C_14_PC_3_NH_3_Cl and their mixtures; the curves correspond to the best fit of Gaussian bilayers or core-shell models. (B) The corresponding electron density profiles of the bilayer models corresponding to the best fits of (A). Figure S44: The core-shell model vesicle model. Figure S45: Hemolysis (%) as a function of surfactants concentration. Figure S46: Three-dimensional (3D) closest interactions between active site residues of peptidoglycan glycosyltransferase (PDB ID:2OQO) with Cn benzalkonium (from C8 to C14 carbon atoms) derivatives. Table S1: Fitting parameters of Gaussian bilayers for DPPC/C_12_PC_3_NH_3_Cl mixtures. Table S2: Fitting parameters of Gaussian bilayers for DPPC/C_14_PC_3_NH_3_Cl mixtures. Table S3 Hemolytic activity of tryptophan and phenylalanine-based surfactants. Table S4: Results of the interaction details and docking score in (kcal/mol) of benzalkonium derivative (from C_8_ to C_14_ carbon atoms) against the peptidoglycan glycosyltransferase (PDB ID:2OQO).
