# Supplementary material for: Phenylalanine and Tryptophan-Based Surfactants as New Antibacterial Agents: Characterization, Self-Aggregation Properties, and DPPC/Surfactants Vesicles Formulation
Source: Pharmaceutics. 2023 Jun 30;15(7):1856. doi: 10.3390/pharmaceutics15071856 (PMC10384047; doi:10.3390/pharmaceutics15071856)
Supplement: Supplementary file 1 [file pharmaceutics-15-01856-s001.zip › pharmaceutics-2426192-supplementary.pdf]

ELECTRONIC SUPPLEMENTARY INFORMATION

Phenylalanine and Tryptophan-based surfactants as new antibacterial agents: characterization, self-aggregation properties, and DPPC/Surfactants vesicles formulation

Zakaria Hafidi[1,2], Lourdes Pérez[1*], Mohammed El Achouri[2,3], Ramon Pons[1*]

[1]Department of Surfactants and Nanobiotechnology, IQAC-CSIC, Barcelona, Spain

[2]Laboratoire de physico-chimie des matériaux inorganiques et organiques, Centre des Sciences des Matériaux, Ecole Normale supérieure-Rabat, Mohammed V University in Rabat, Rabat, Morocco

[3] Centre des Sciences et Technologies de la Formulation

CONTENT

Figures

Figure. S1: ^{1}H NMR spectrum of $C_8TC_3NH_3C$

Figure. S2: ^{13}C NMR spectrum of $C_8TC_3NH_3Cl$

Figure. S3: ESI-MS spectrum of $C_8TC_3NH_3Cl$

Figure. S4: ^{1}H NMR spectrum of $C_{10}TC_3NH_3C$

Figure. S5 ^{13}C NMR spectrum of $C_{10}TC_3NH_3C$

Figure. S6: ESI-MS spectrum of $C_{10}TC_3NH_3Cl$

Figure. S7: ^{1}H NMR spectrum of $C_{12}TC_3NH_3C$

Figure. S8: ^{1}H NMR spectrum of $C_{12}TC_3NH$

Figure. S9: ESI-MS spectrum of $C_{12}TC_3NH_3Cl$

Figure. S10: ^{1}H NMR spectrum of $C_{14}TC_3NH_3C$

Figure. S11: ^{13}C NMR spectrum of $C_{12}TC_3NH$

Figure. S12: ESI-MS spectrum of $C_{14}TC_3NH_3Cl$

Figure. S13: ^{1}H NMR spectrum of $C_{10}PC_3NH_3C$

Figure. S14: ^{13}C NMR spectrum of $C_{10}PC_3NH_3C$

Figure. S15: ESI-MS spectrum of $C_{10}PC_3NH_3C$

Figure. S16: ^{1}H NMR spectrum of $C_{12}PC_3NH_3C$

Figure. S17: ^{13}C NMR spectrum of $C_{12}PC_3NH_3C$

Figure. S18: ESI-MS spectrum of $C_{12}PC_3NH_3C$

Figure. S19: ^{1}H NMR spectrum of $C_{14}PC_3NH_3C$

Figure. S20: ^{13}C NMR spectrum of $C_{14}PC_3NH_3C$
Figure. S21: ESI-MS spectrum of $C_{14}PC_3NH_3C$

Figure.S22 NaOH titration and HCl back titration at 298.15 K for $C_8TC_3NH_3Cl$
Figure.S23 NaOH titration and HCl back titration at 298.15 K for $C_{10}TC_3NH_3Cl$
Figure.S24 NaOH titration and HCl back titration at 298.15 K for $C_{12}TC_3NH_3Cl$
Figure.S25 NaOH titration and HCl back titration at 298.15 K for $C_{14}TC_3NH_3Cl$
Figure.S26 NaOH titration and HCl back titration at 298.15 K for $C_{10}PC_3NH_3Cl$
Figure.S27 NaOH titration and HCl back titration at 298.15 K for $C_{12}PC_3NH_3Cl$
Figure.S28 NaOH titration and HCl back titration at 298.15 K for $C_{14}PC_3NH_3Cl$
Figure.S29 Specific conductivity (κ) as a function of $C_{10}TC_3NH_3Cl$ concentration
Figure.S30 Specific conductivity (κ) as a function of $C_{12}TC_3NH_3Cl$ concentration
Figure.S31 Specific conductivity (κ) as a function of $C_{14}TC_3NH_3Cl$ concentration
Figure.S32 Specific conductivity (κ) as a function of $C_{10}PC_3NH_3Cl$ concentration
Figure.S33 Specific conductivity (κ) as a function of $C_{12}PC_3NH_3Cl$ concentration
Figure.S34 Specific conductivity (κ) as a function of $C_{14}PC_3NH_3Cl$ concentration
Figure.S35 Variation of I_1/I_3 ratio of pyrene fluorescence as a function of $C_8TC_3NH_3Cl$ concentration
Figure.S36 Variation of I_1/I_3 ratio of pyrene fluorescence as a function of $C_{10}TC_3NH_3Cl$ concentration
Figure.S37 Variation of I_1/I_3 ratio of pyrene fluorescence as a function of $C_{12}TC_3NH_3Cl$ concentration
Figure.S38 Variation of I_1/I_3 ratio of pyrene fluorescence as a function of $C_{14}TC_3NH_3Cl$concentration
Figure.S39 Variation of I_1/I_3 ratio of pyrene fluorescence as a function of $C_{14}PC_3NH_3Cl$ concentration
Figure.S40 Variation of I_1/I_3 ratio of pyrene fluorescence as a function of $C_{12}PC_3NH_3Cl$ concentration
Figure.S41 Variation of I_1/I_3 ratio of pyrene fluorescence as a function of $C_{14}PC_3NH_3Cl$ concentration
Figure.S42 A) Scattered intensity patterns as a function of scattering vector modulus for DPPC and $C_{12}PC_3NH_3Cl$ and their mixtures the curves correspond to the best fit of Gaussian bilayers or core-shell models. B) The corresponding electron density profiles of the bilayer models corresponding to the best fits of A).

Figure.S43 A) Scattered intensity patterns as a function of scattering vector modulus for DPPC and $C_{14}PC_3NH_3Cl$ and their mixtures the curves correspond to the best fit of Gaussian bilayers or core-shell models. B) The corresponding electron density profiles of the bilayer models corresponding to the best fits of A).

Figure.S44. the core-shell model vesicle model

Figure. S45. Hemolysis (%) as a function of surfactants concentration.

Figure S46: Three-dimensional (3 D) closest interactions between active site residues of peptidoglycan glycosyltransferase (PDB ID:2OQO) With CnBenzalkonium (From C_8 to C_{14} carbon atoms) derivatives.

Tables

Table S1 Fitting parameters of Gaussian bilayers for DPPC/$C_{12}PC_3NH_3Cl$ mixtures.
Table S2 Fitting parameters of Gaussian bilayers for DPPC/$C_{14}PC_3NH_3Cl$ mixtures
Table S3 Hemolytic activity of tryptophan and phenylalanine-based surfactants
Table S4 Results of the interaction details and docking score in (kcal/mol) of Benzalkonium derivative (From C8 to C14 carbon atoms) against the peptidoglycan glycosyltransferase (PDB ID:2OQO).

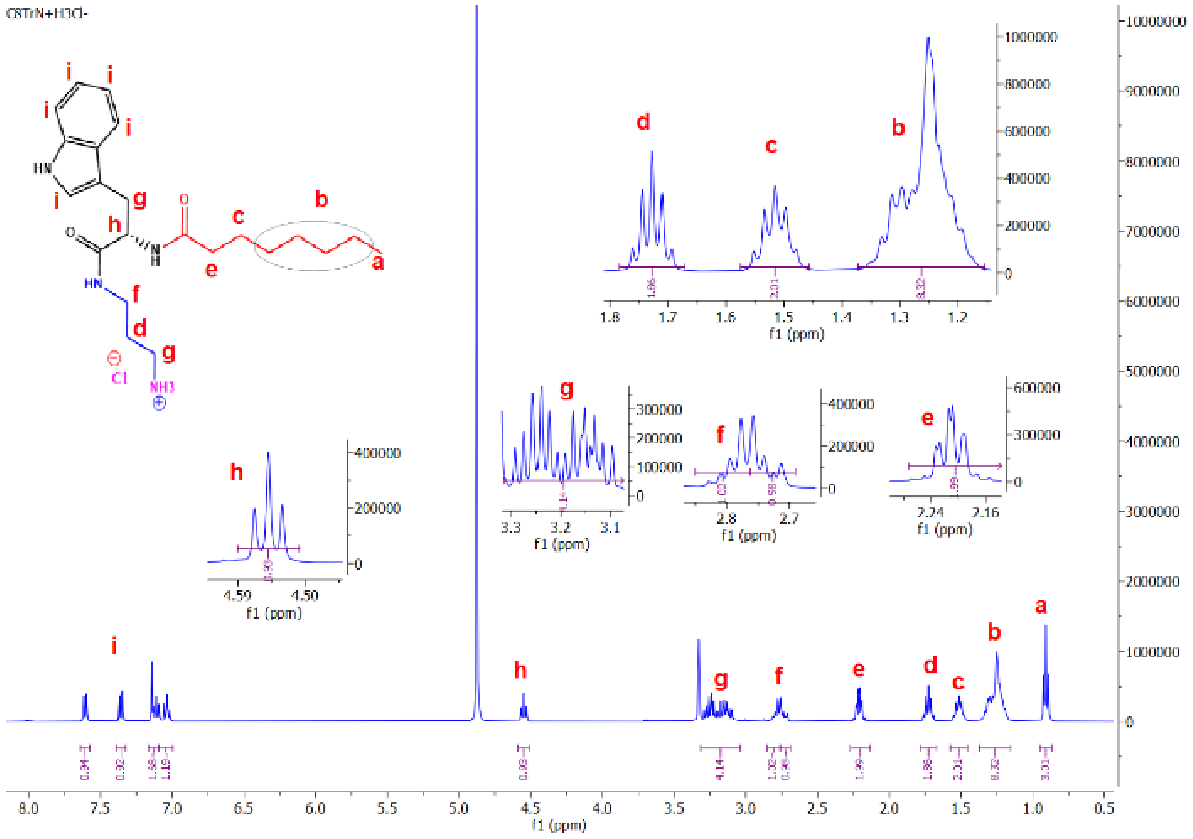

Figure. S1: ^{1}H NMR spectrum of $C_8TC_3NH_3C$

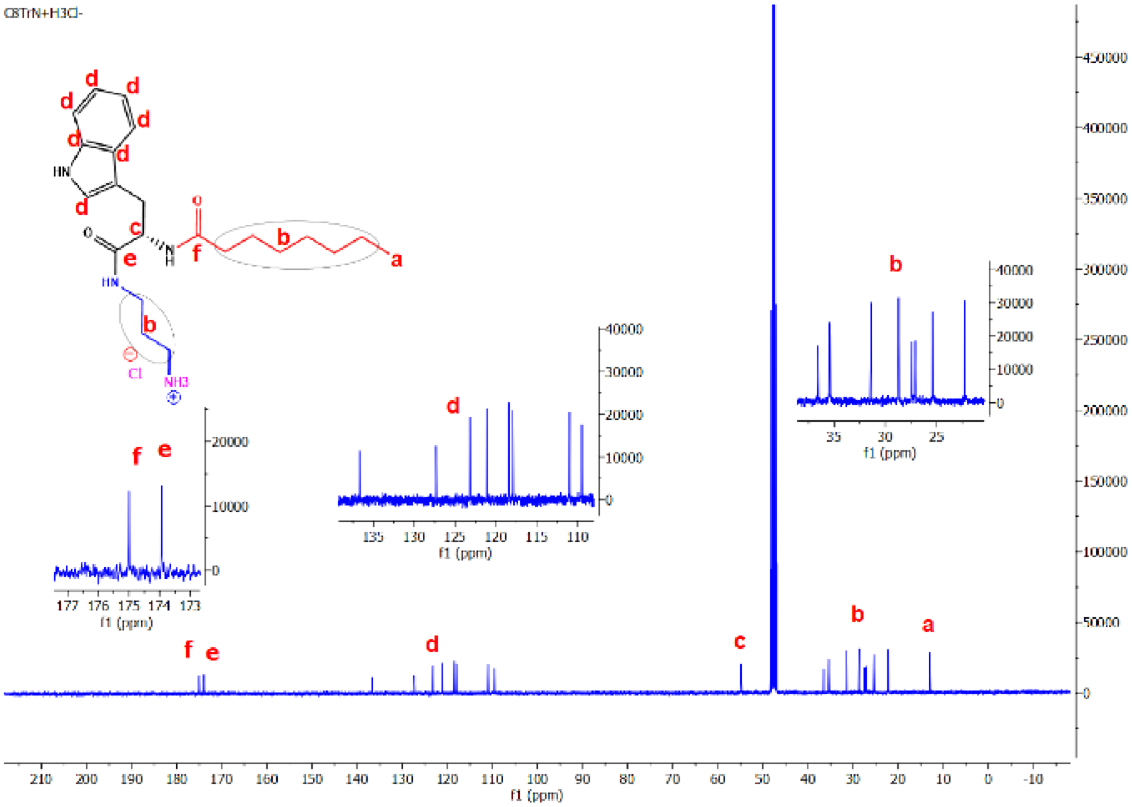

Figure. S2: ^{13}C NMR spectrum of $C_8TC_3NH_3Cl$

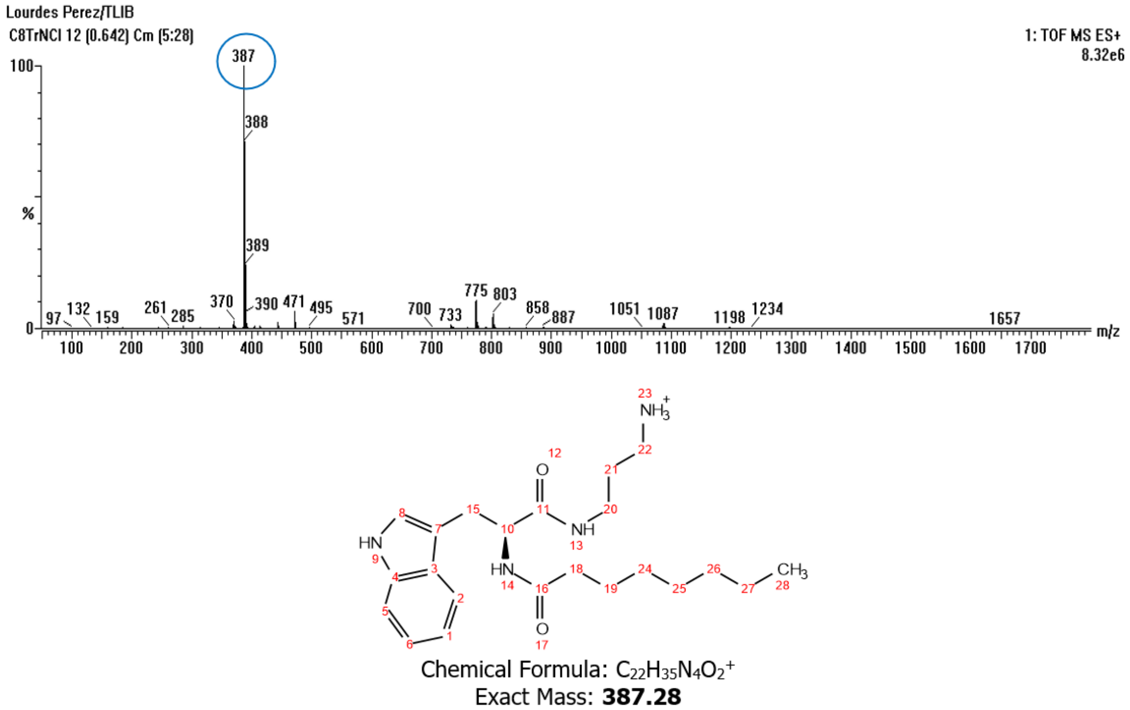

Figure. S3: ESI-MS spectrum of $C_8TC_3NH_3Cl$

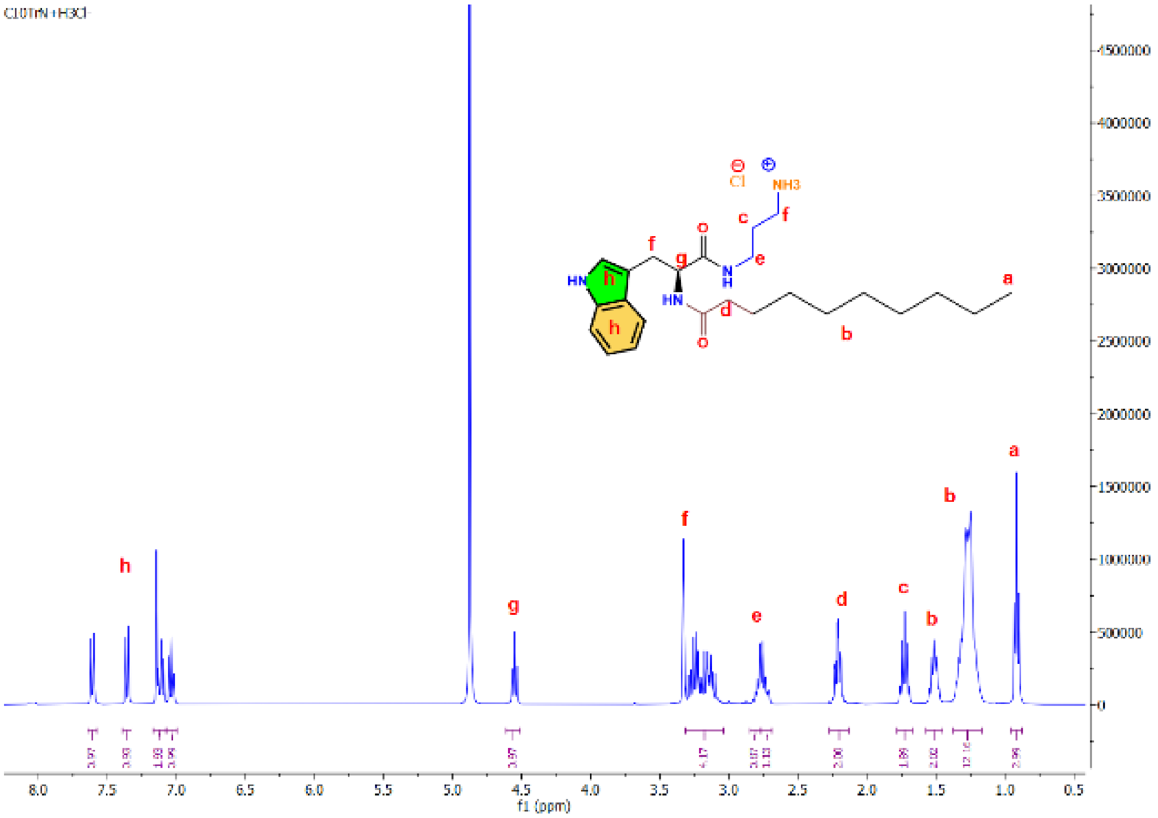

Figure. S4: ^{1}H NMR spectrum of $C_{10}TC_{3}NH_{3}C$

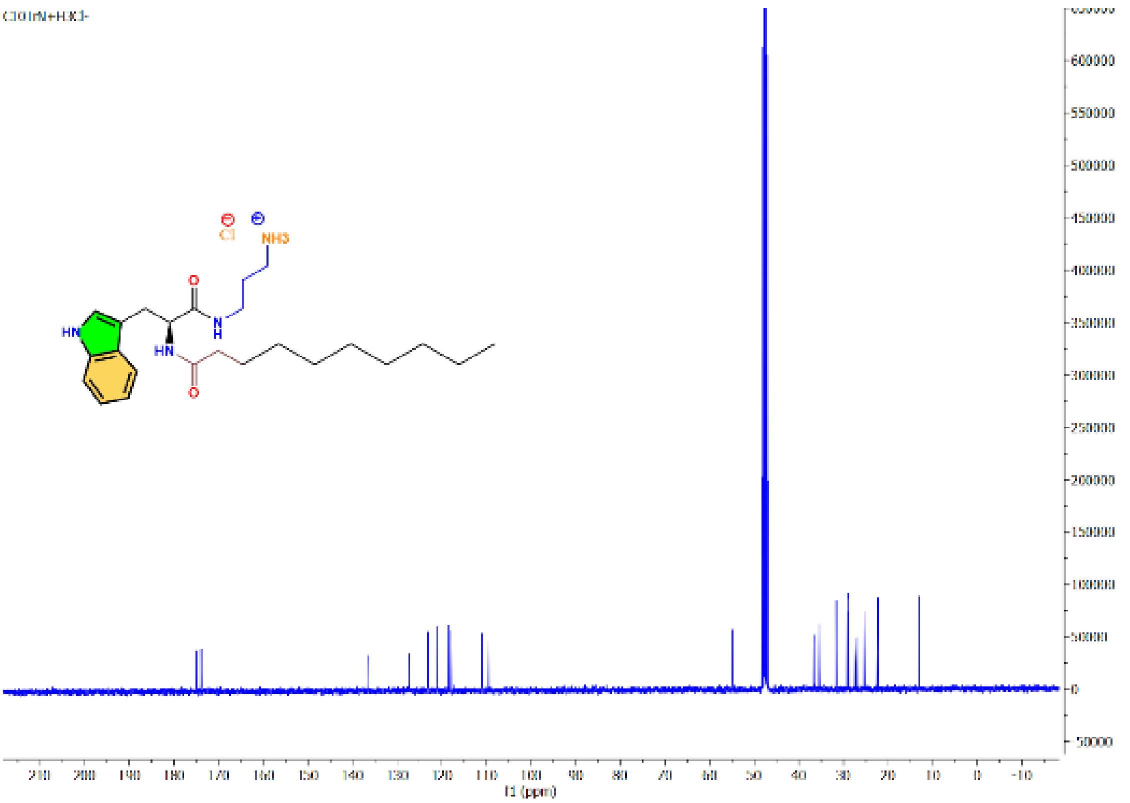

Figure. S5 ^{13}C NMR spectrum of $C_{10}TC_3NH_3C$

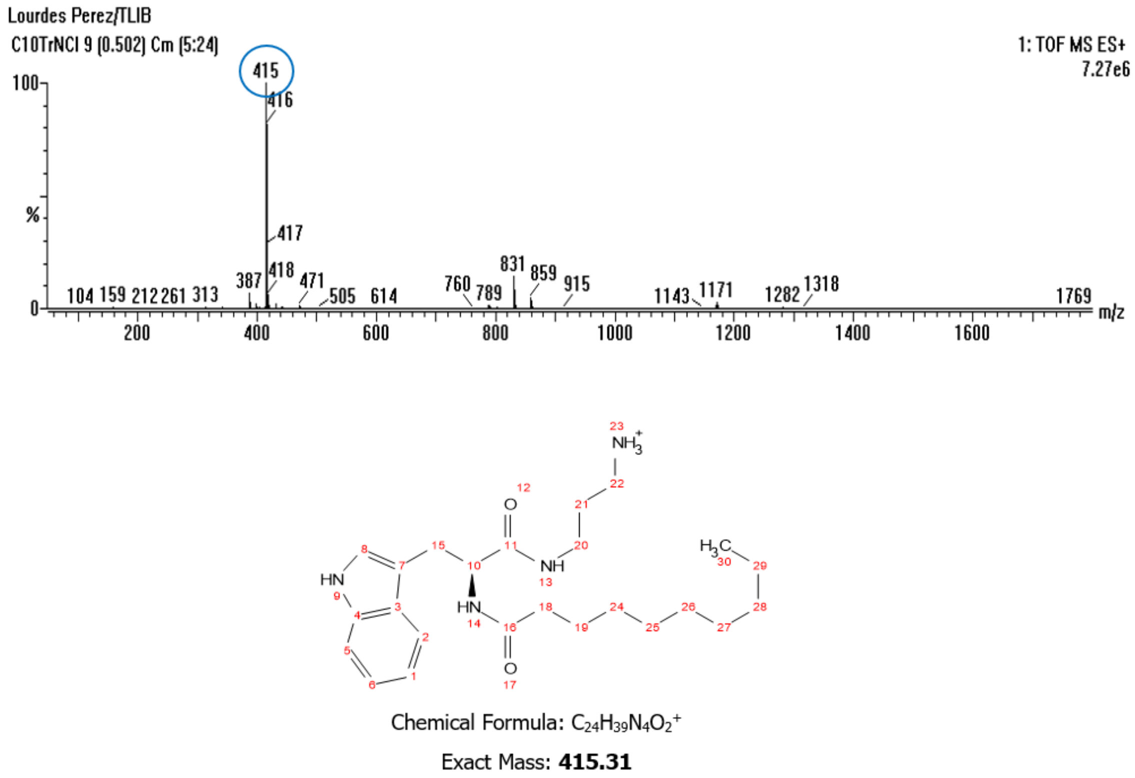

Figure. S6: ESI-MS spectrum of $C_{10}TC_3NH_3Cl$

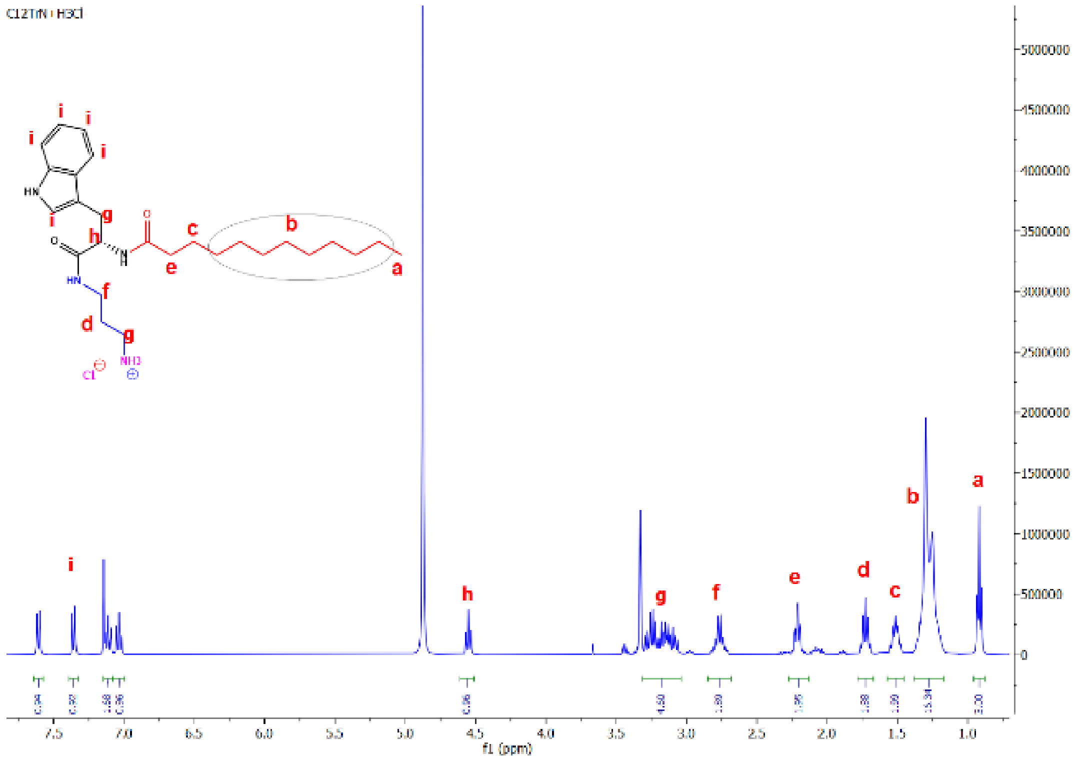

Figure. S7: ^{1}H NMR spectrum of $C_{12}TC_3NH_3C$

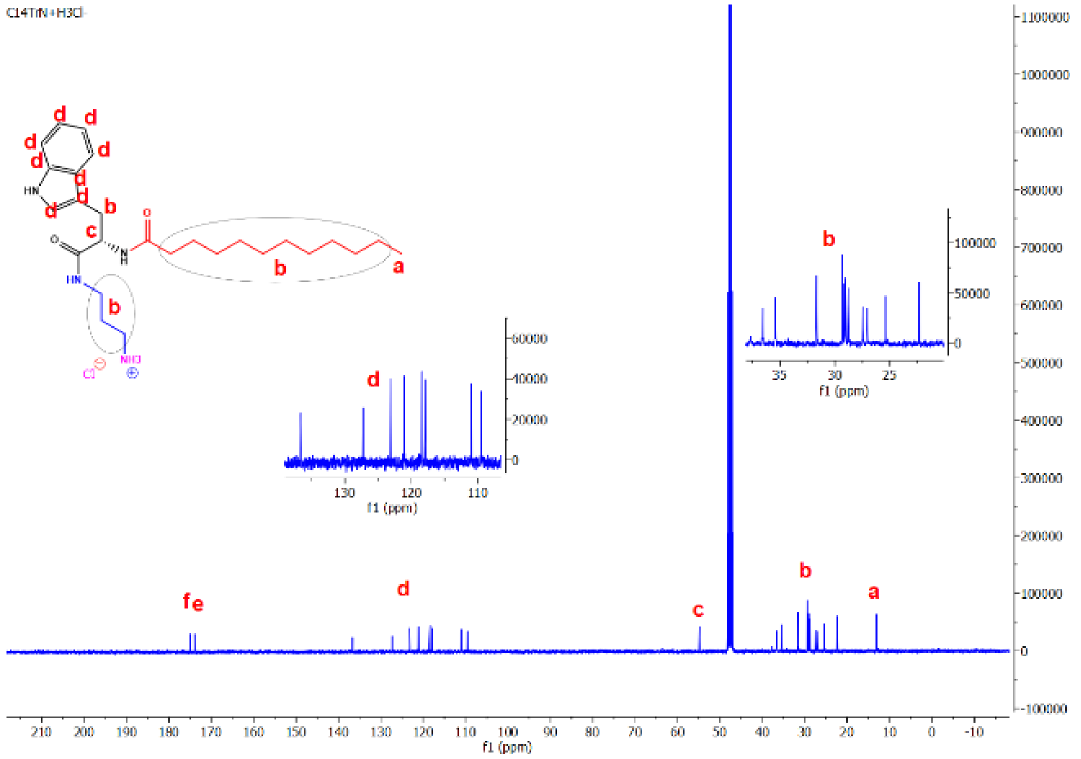

Figure. S8: ^{1}H NMR spectrum of $C_{12}TC_3NH$

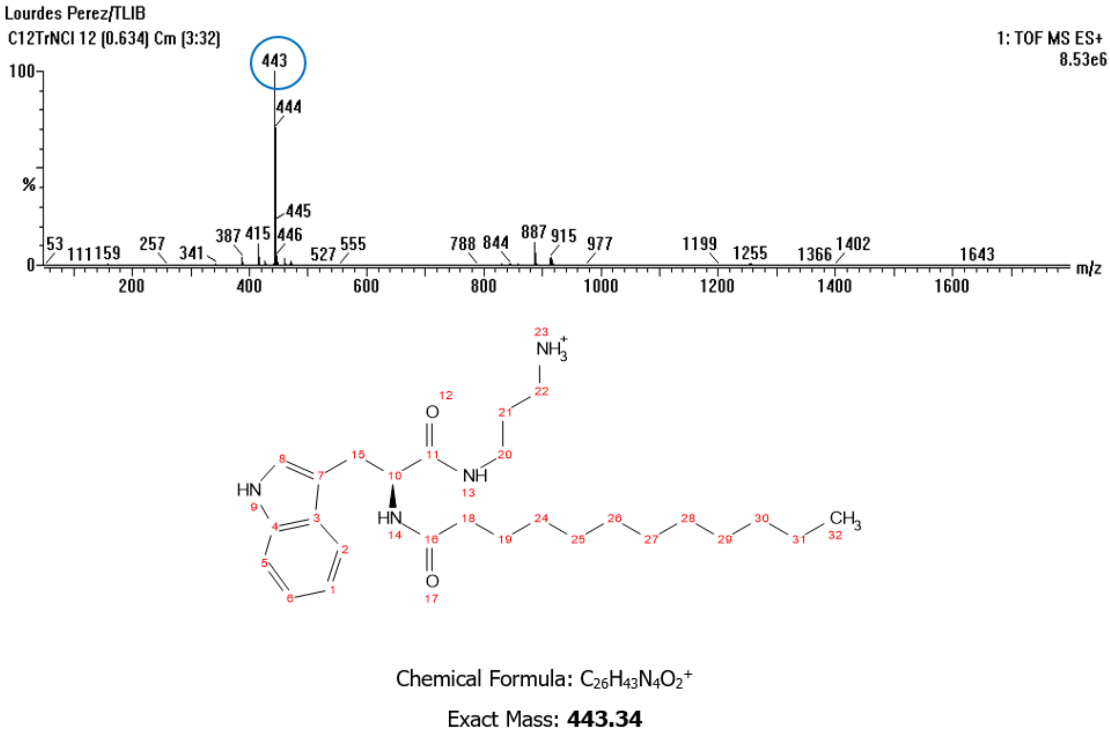

Figure. S9: ESI-MS spectrum of $C_{12}TC_3NH_3Cl$

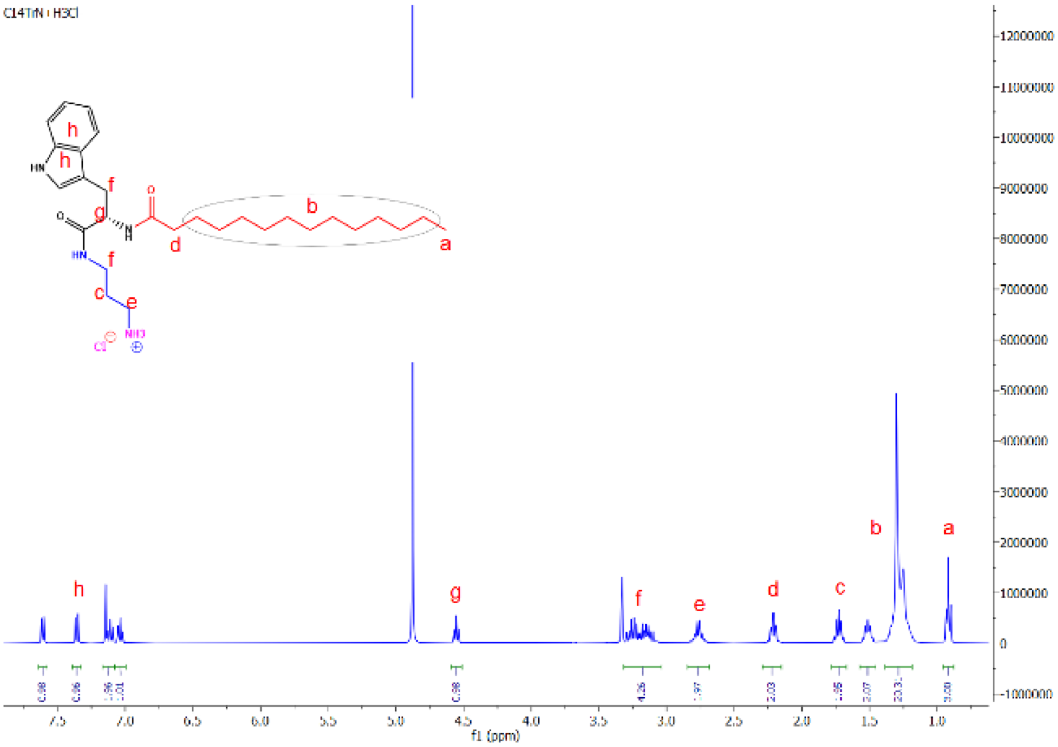

Figure. S10: ^{1}H NMR spectrum of $C_{14}TC_{3}NH_{3}C$

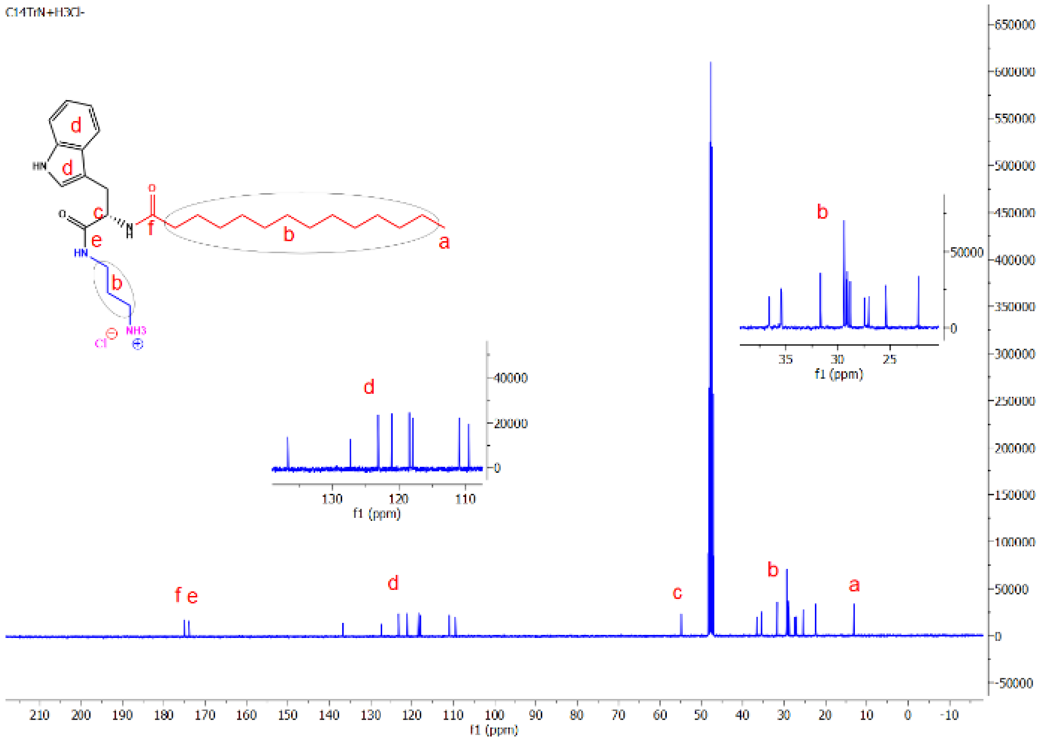

Figure. S11: ^{13}C NMR spectrum of $C_{12}TC_{3}NH$

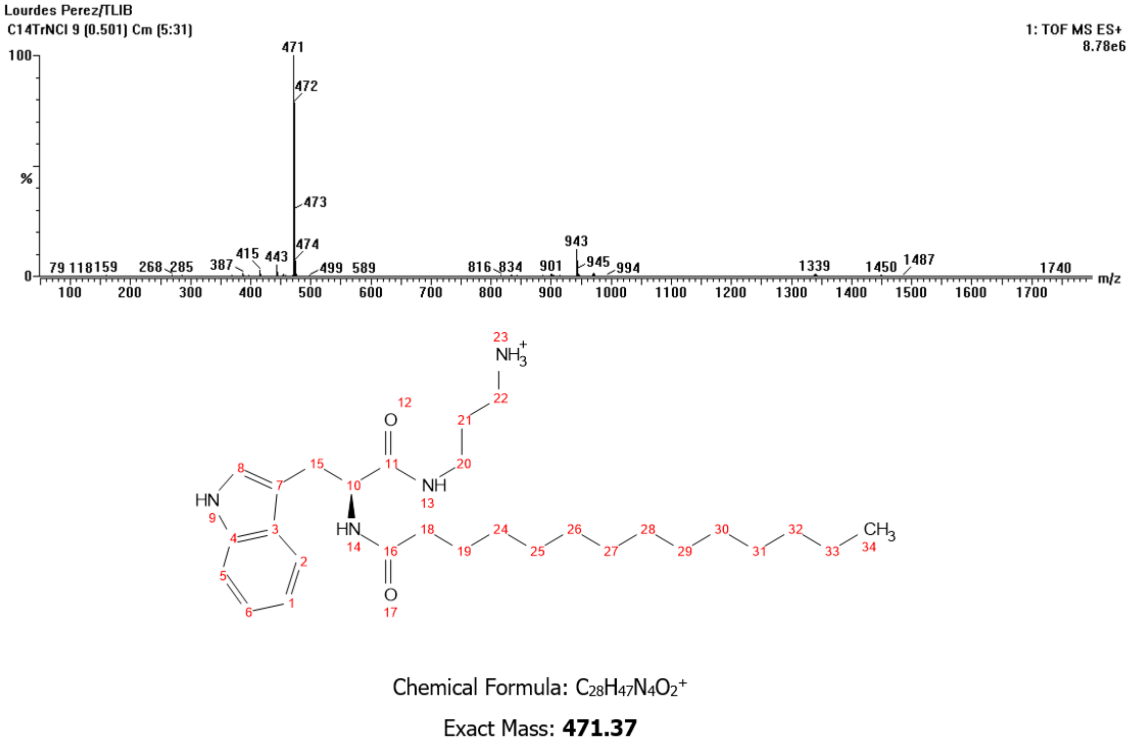

Figure. S12: ESI-MS spectrum of $C_{14}TC_3NH_3Cl$

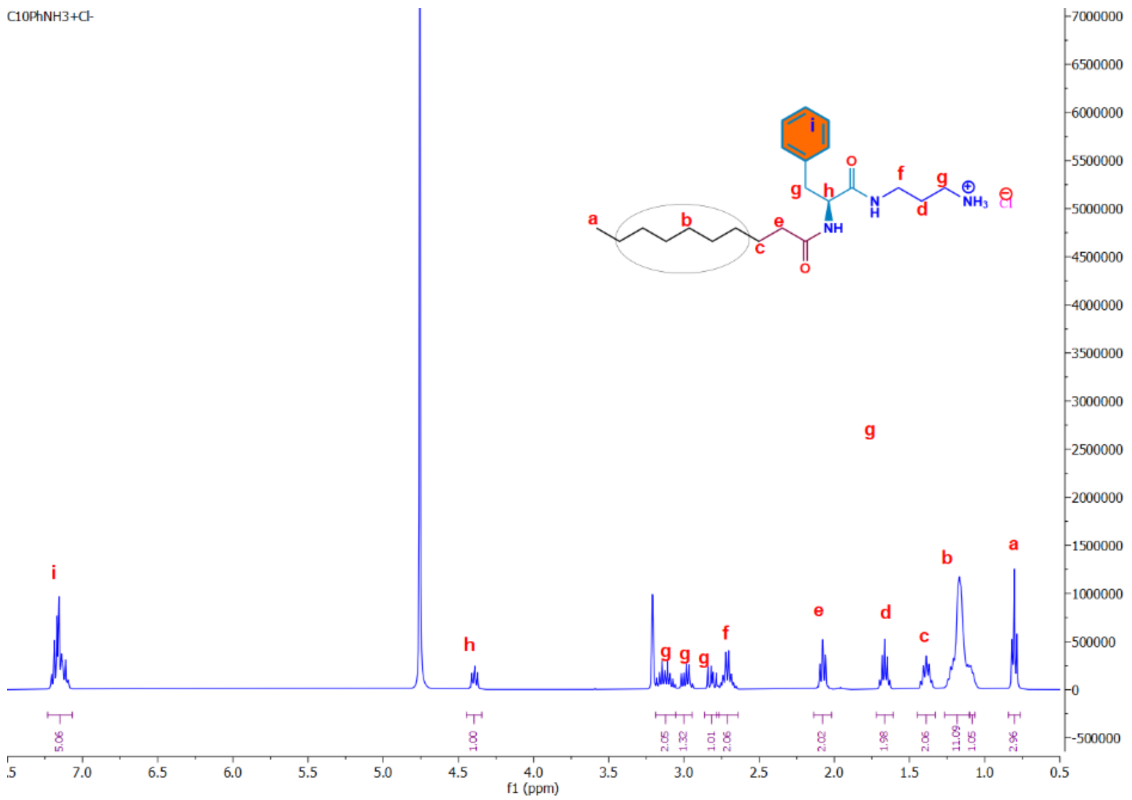

Figure. S13: ^{1}H NMR spectrum of $C_{10}PC_{3}NH_{3}C$

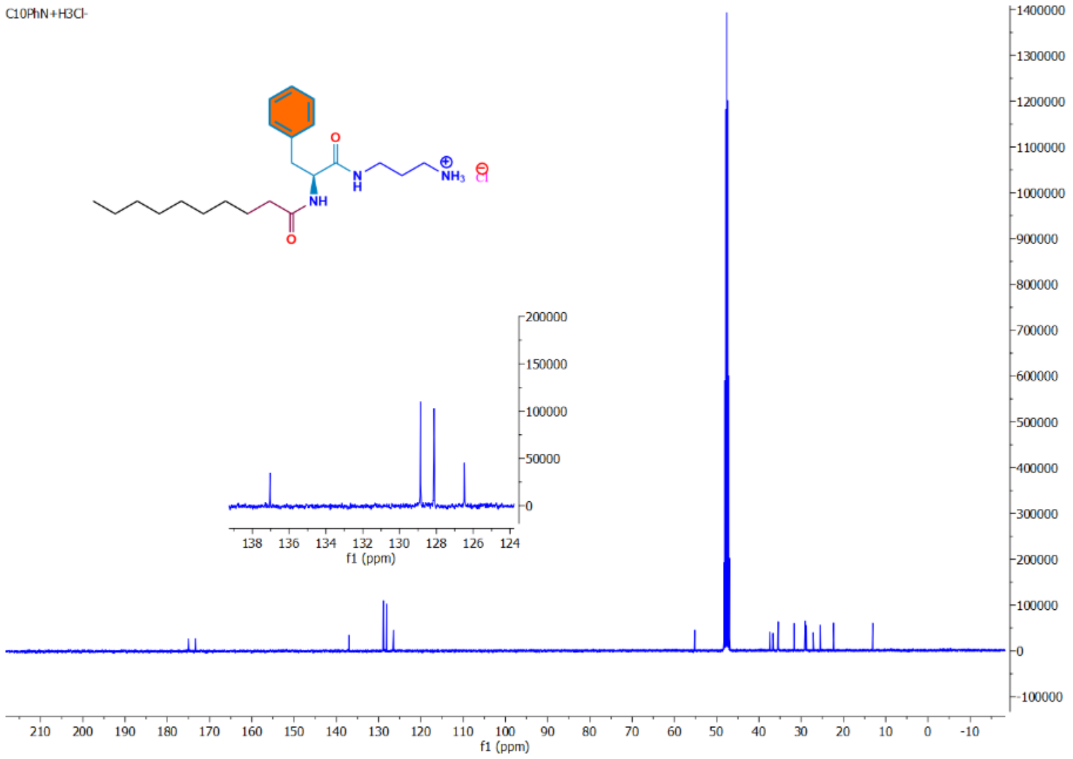

Figure. S14: ^{13}C NMR spectrum of $C_{10}PC_{3}NH_{3}C$

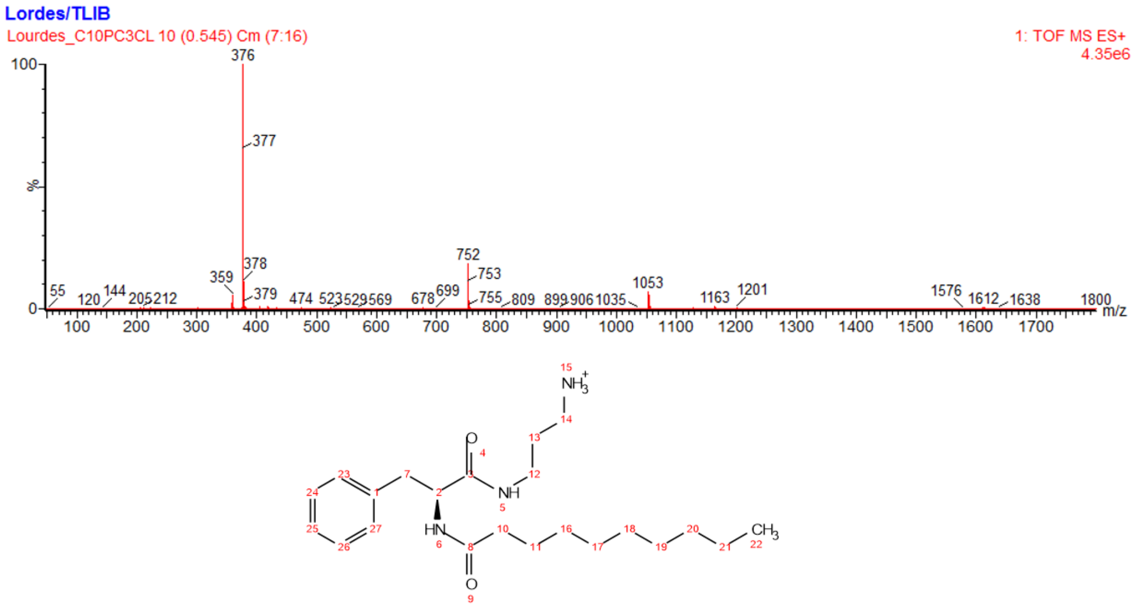

Chemical Formula: $C_{22}H_{38}N_3O_2^+$

Exact Mass: **376.30**

Figure. S15: ESI-MS spectrum of $C_{10}PC_3NH_3C$

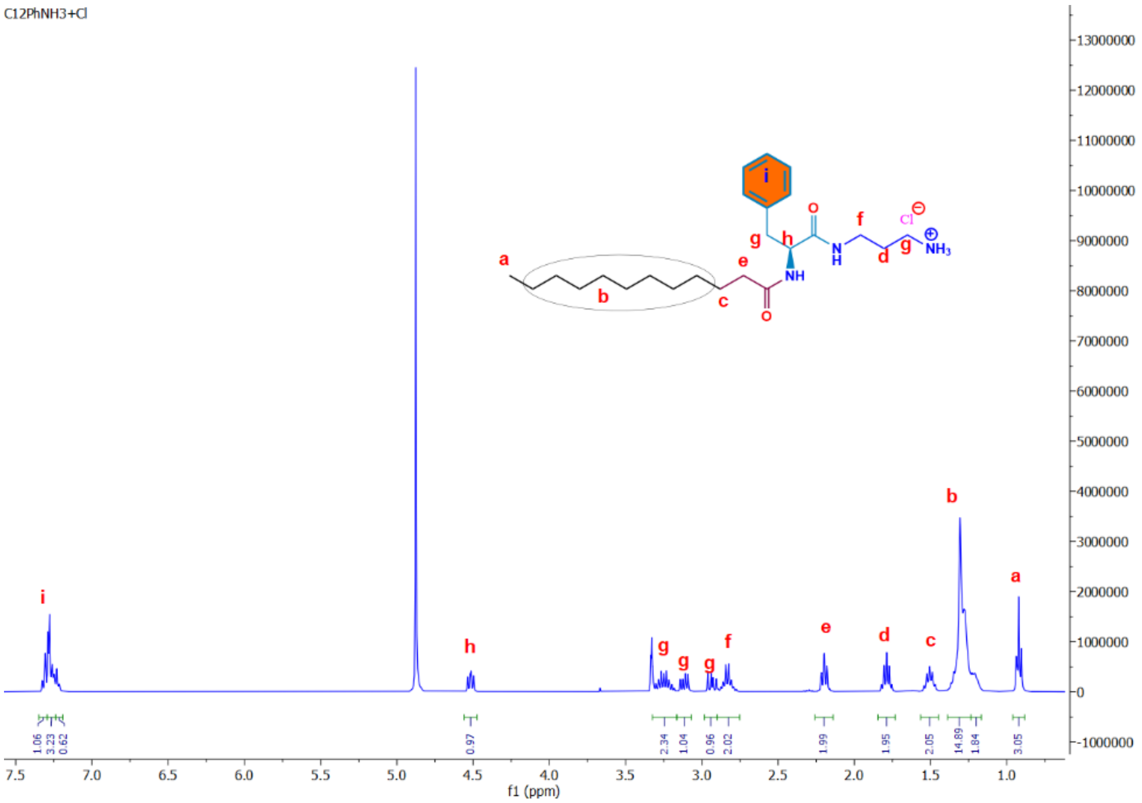

Figure. S16: ^{1}H NMR spectrum of $C_{12}PC_3NH_3C$

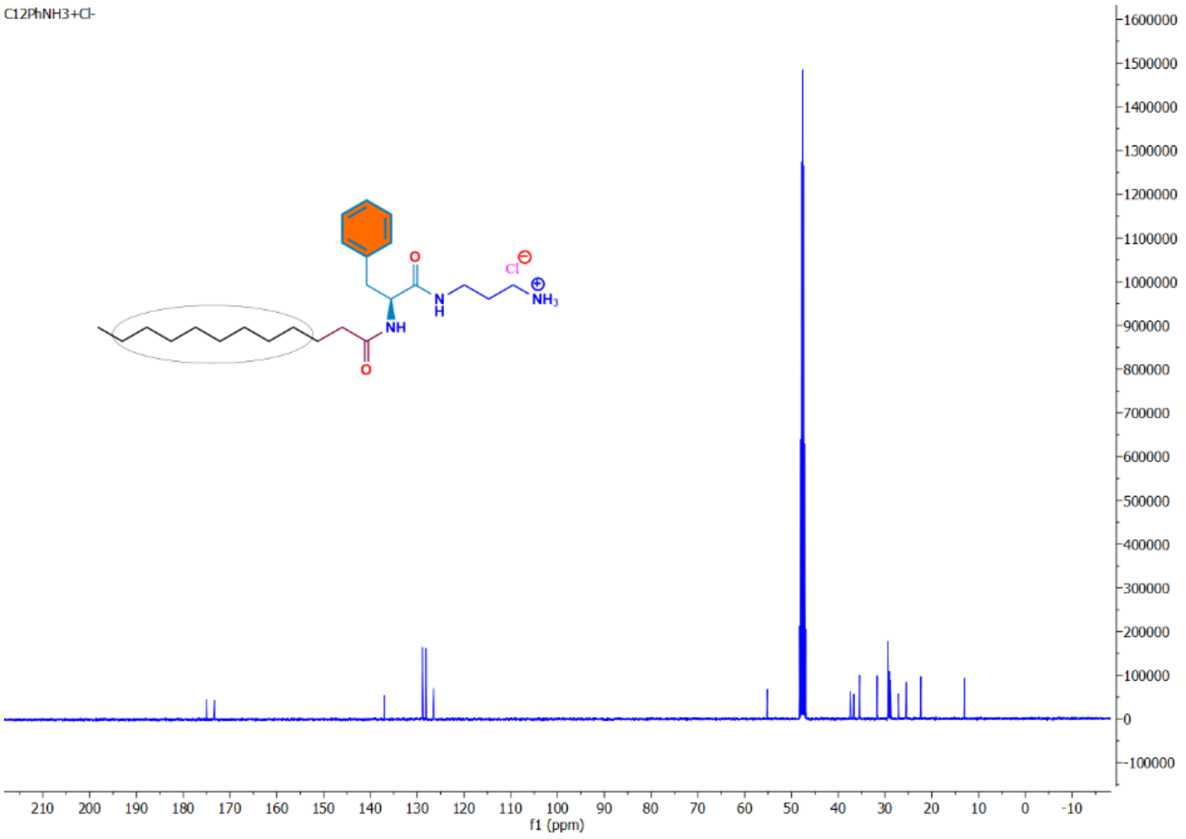

Figure. S17: ^{13}C NMR spectrum of $C_{12}PC_3NH_3C$

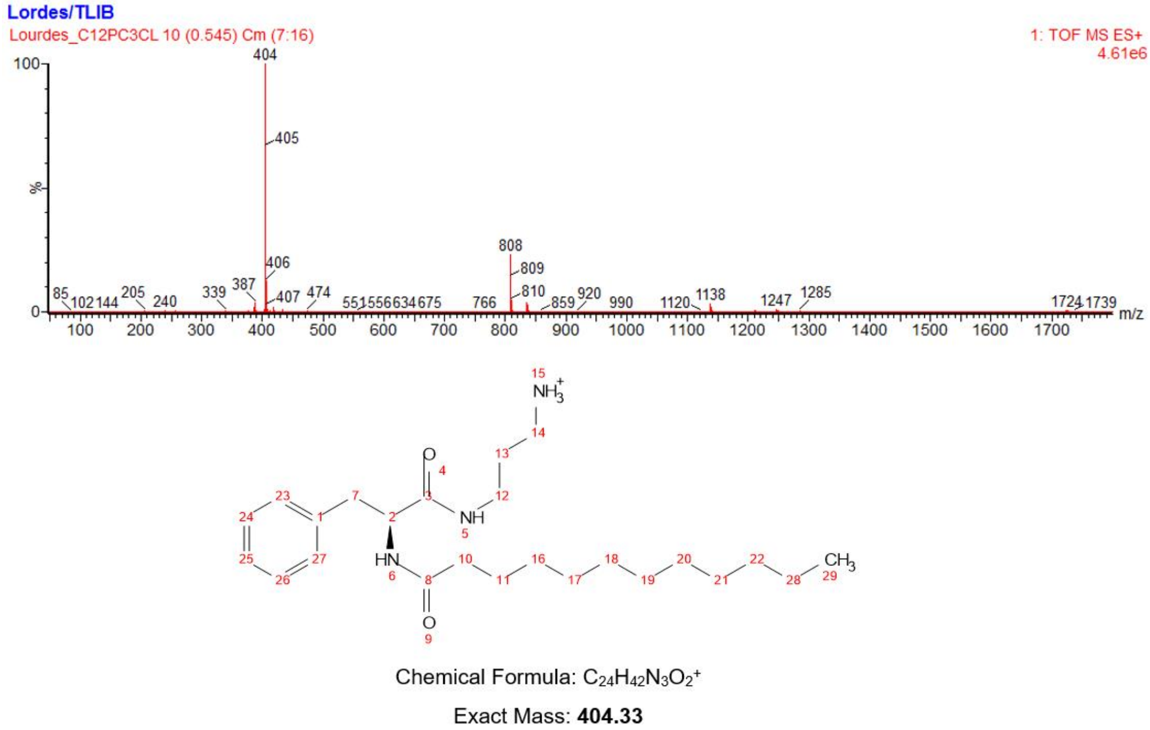

Figure. S18: ESI-MS spectrum of $C_{12}PC_3NH_3C$

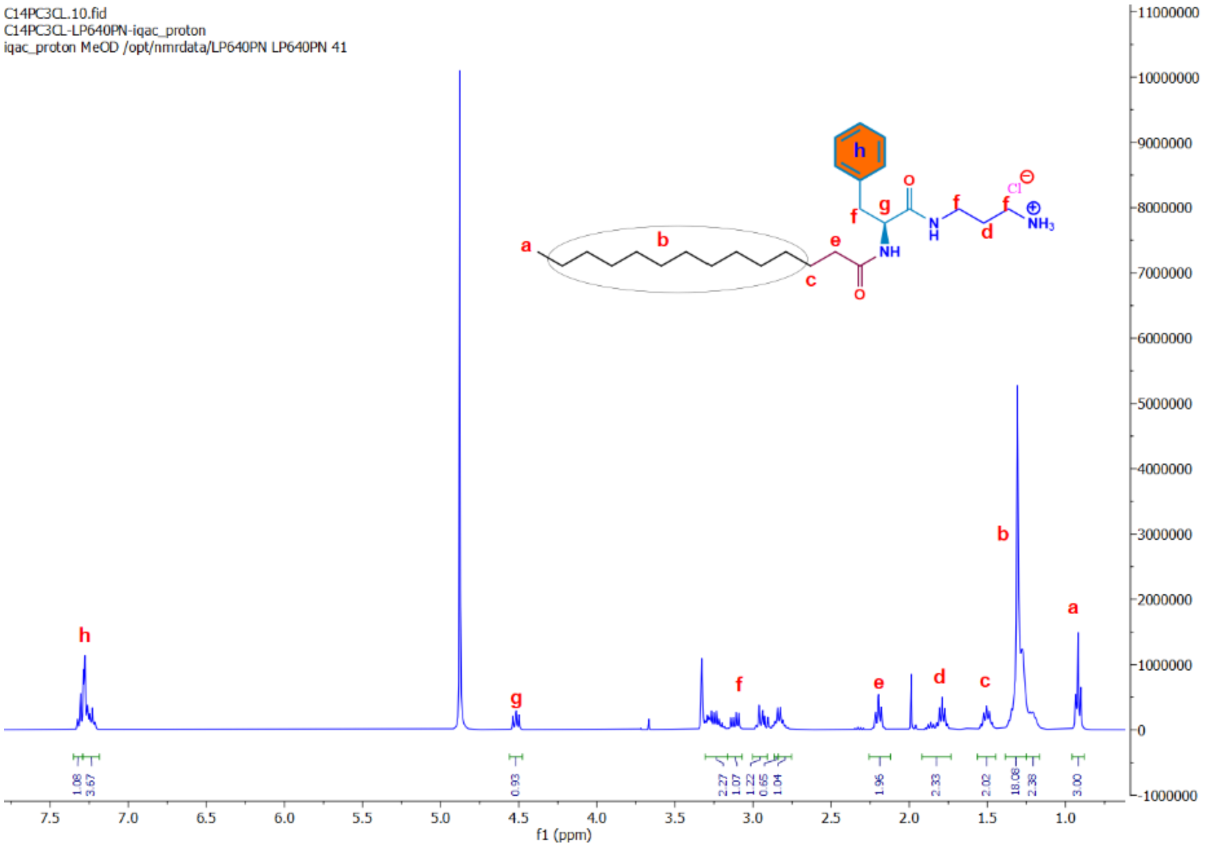

Figure. S19: ^{1}H NMR spectrum of $C_{14}PC_3NH_3C$

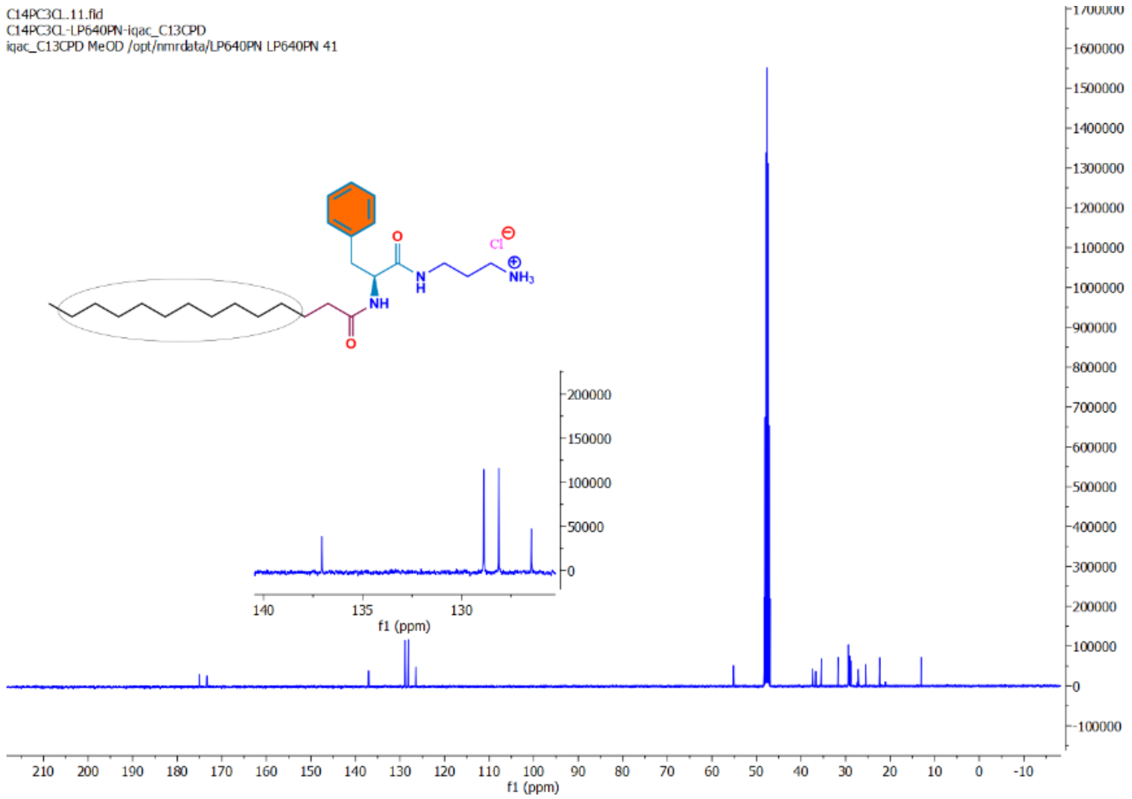

Figure. S20: ^{13}C NMR spectrum of $C_{14}PC_3NH_3C$

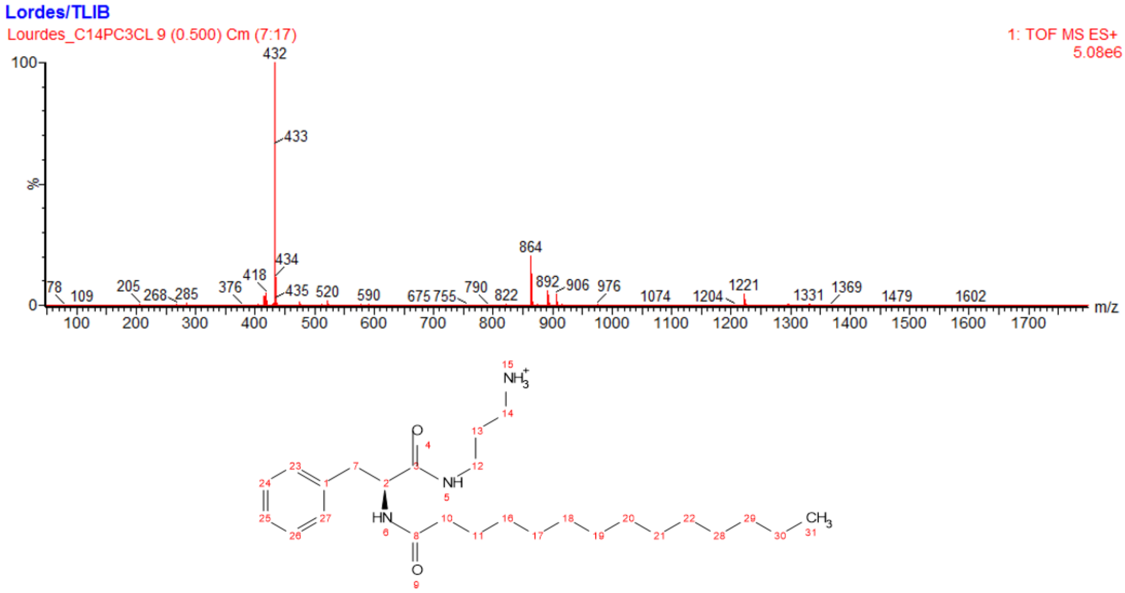

Chemical Formula: $C_{26}H_{46}N_3O_2^+$

Exact Mass: **432.36**

Figure. S21: ESI-MS spectrum of $C_{14}PC_3NH_3C$

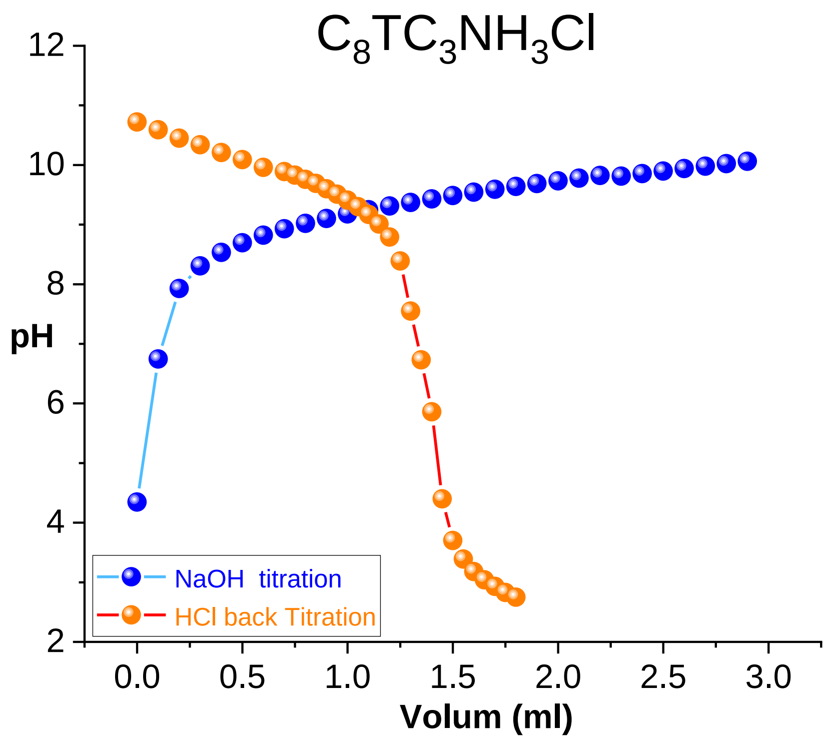

Figure.S22 NaOH titration and HCl backtitration at 298.15 K for $C_8TC_3NH_3Cl$

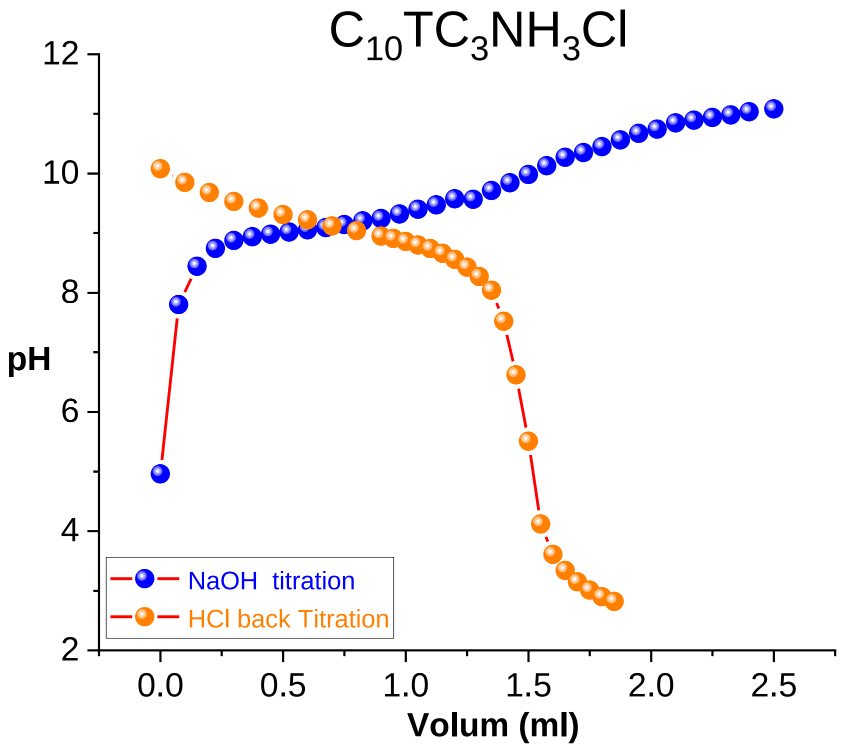

Figure.S23 NaOH titration and HCl backtitration at 298.15 K for $C_{10}TC_3NH_3Cl$

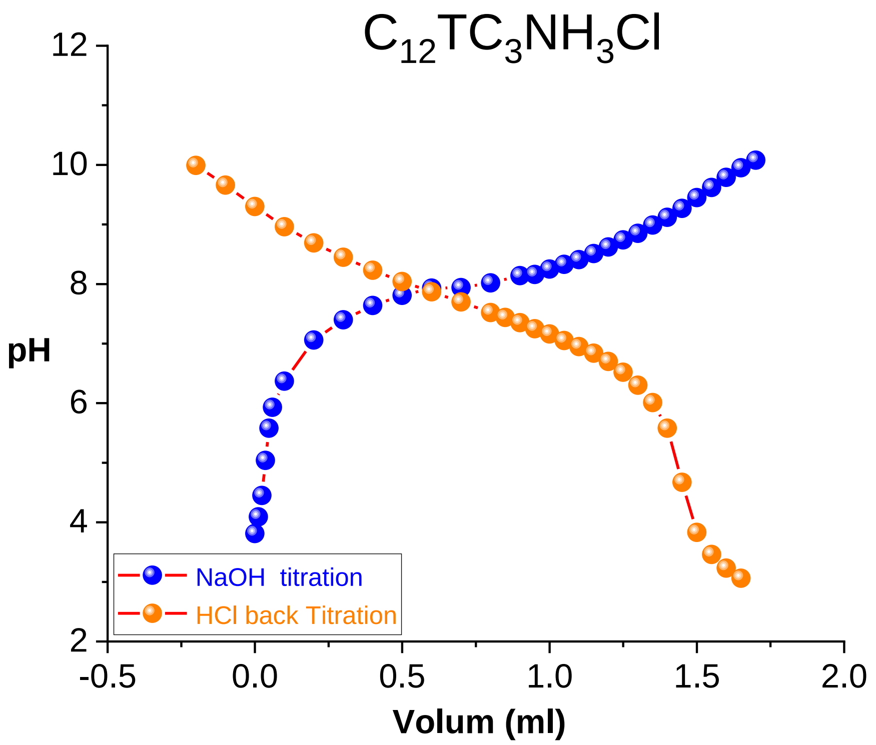

Figure.S24 NaOH titration and HCl back titration at 298.15 K for $C_{12}TC_3NH_3Cl$

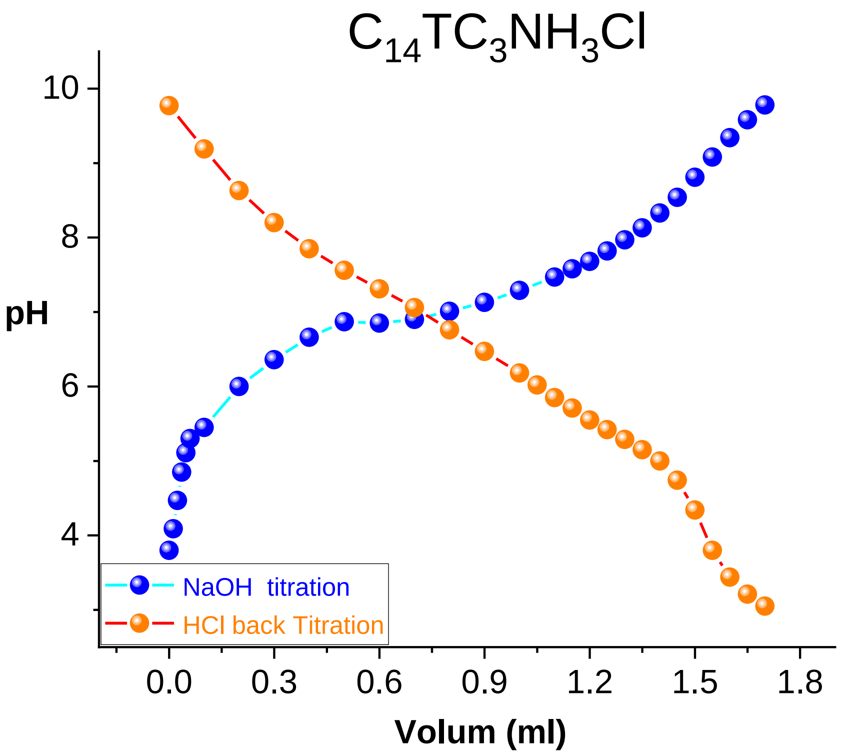

Figure.S25 NaOH titration and HCl backtitration at 298.15 K for $C_{14}TC_3NH_3Cl$

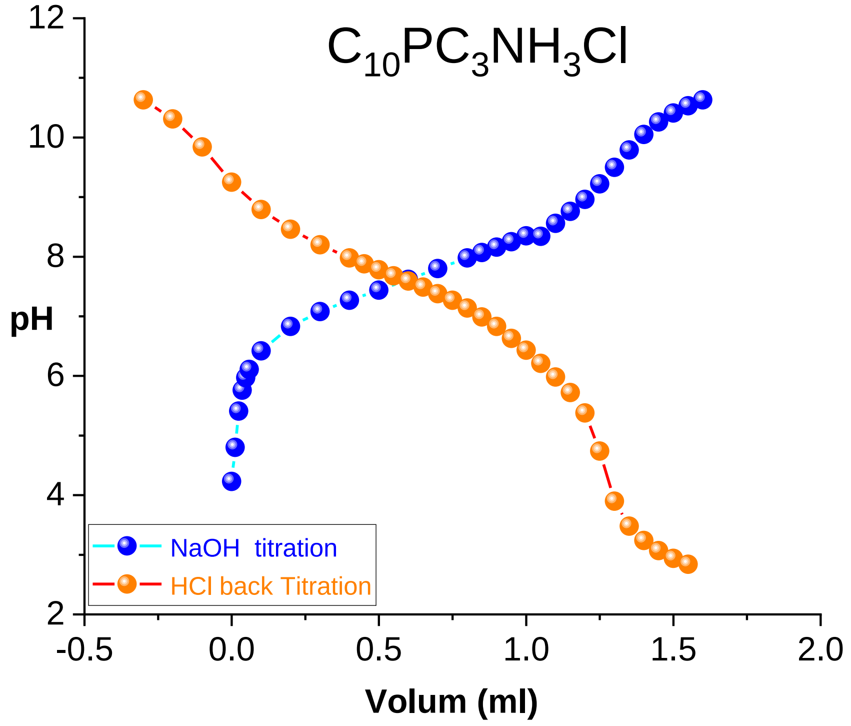

Figure.S26 NaOH titration and HCl backtitration at 298.15 K for $C_{10}PC_3NH_3Cl$

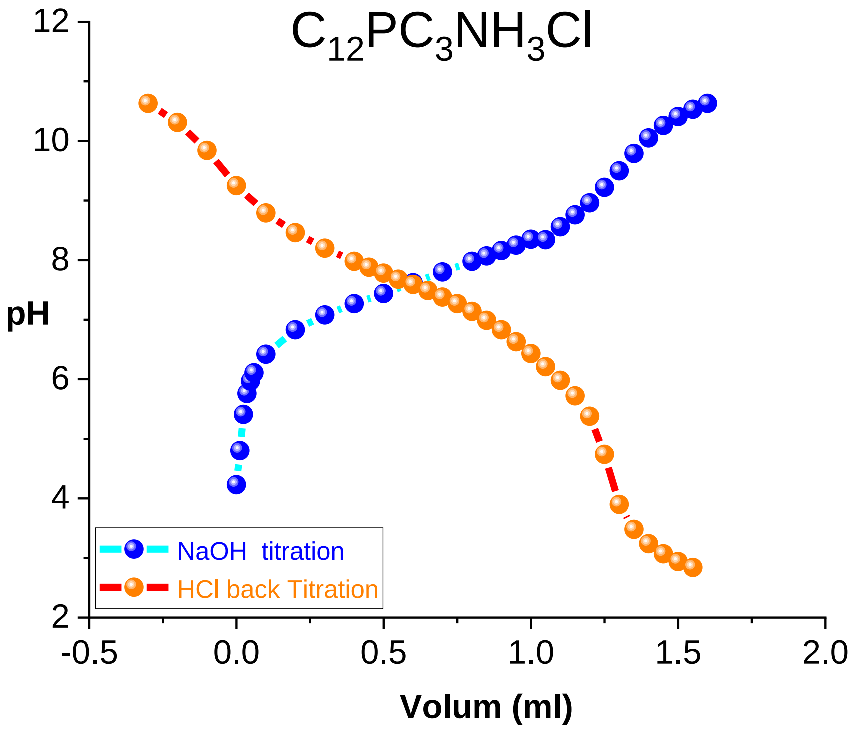

Figure.S27 NaOH titration and HCl back titration at 298.15 K for $C_{12}PC_3NH_3Cl$

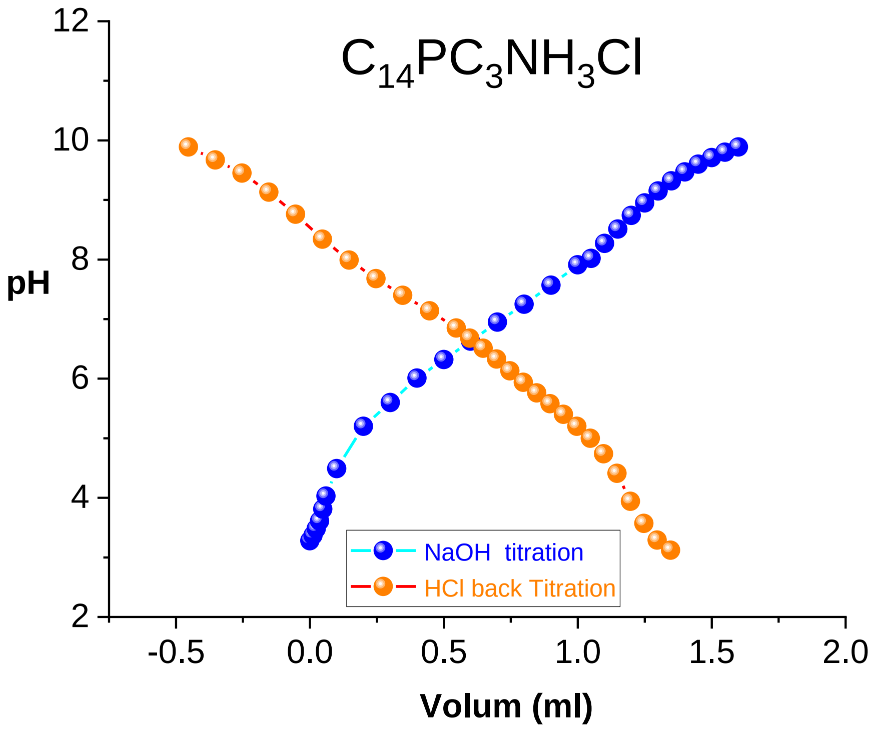

Figure.S28 NaOH titration and HCl backtitration at 298.15 K for $C_{14}PC_3NH_3Cl$

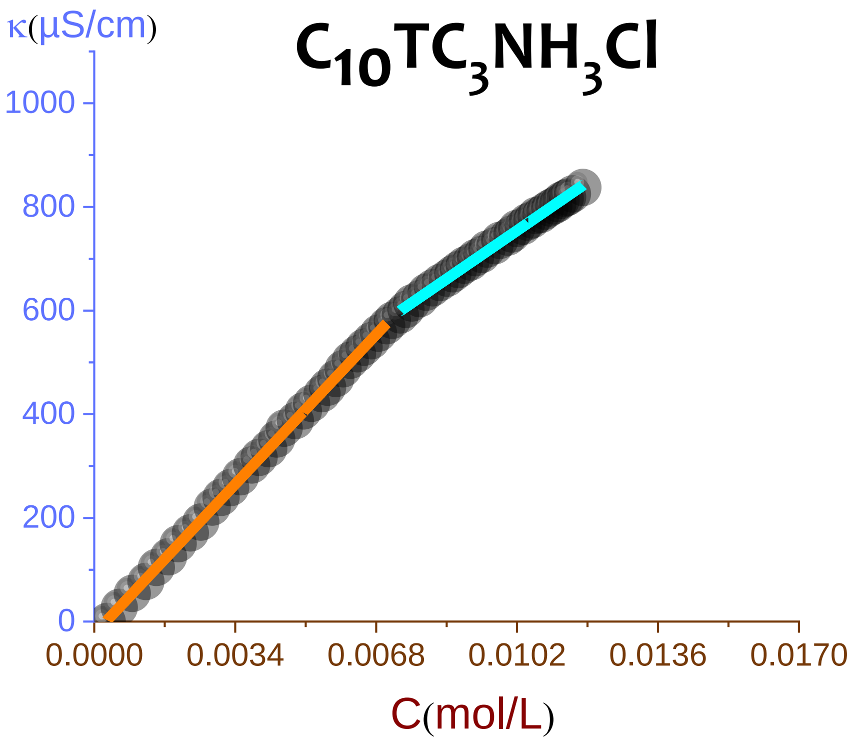

Figure.S29 Specific conductivity (κ) as a function of concentration of $C_{10}TC_3NH_3Cl$

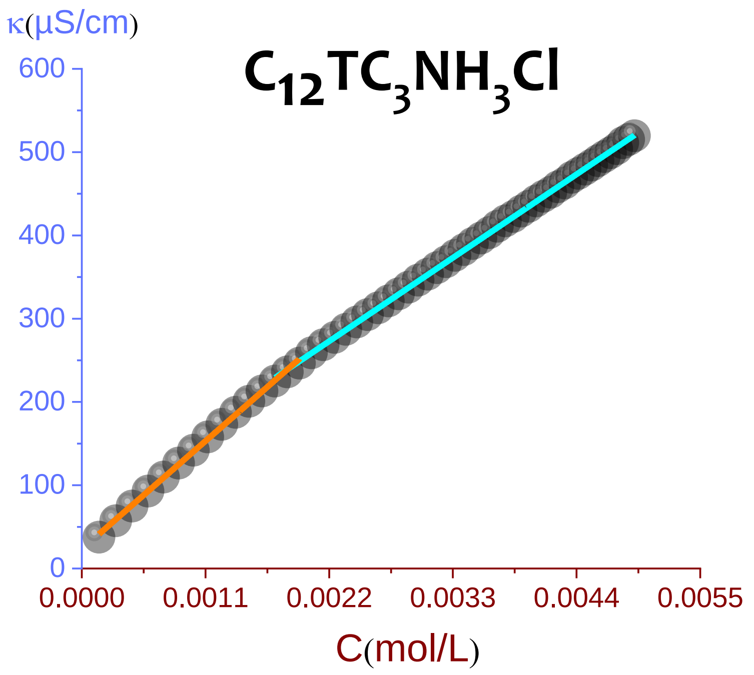

Figure.S30 Specific conductivity (κ) as a function of concentration of $C_{12}TC_3NH_3Cl$

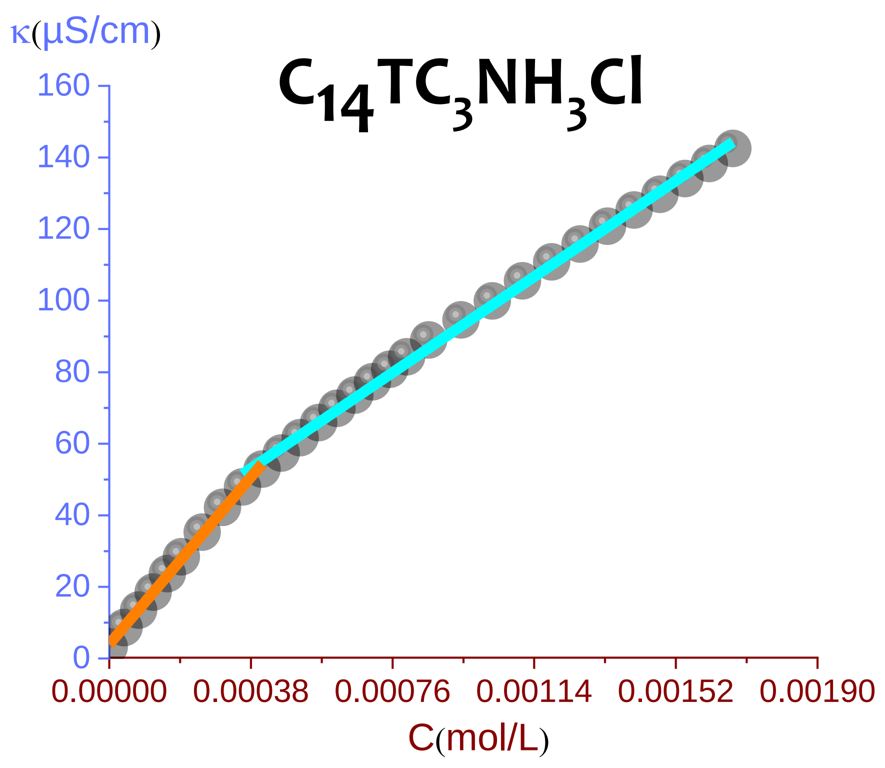

Figure.S31 Specific conductivity (κ) as a function of concentration of $C_{14}TC_3NH_3Cl$

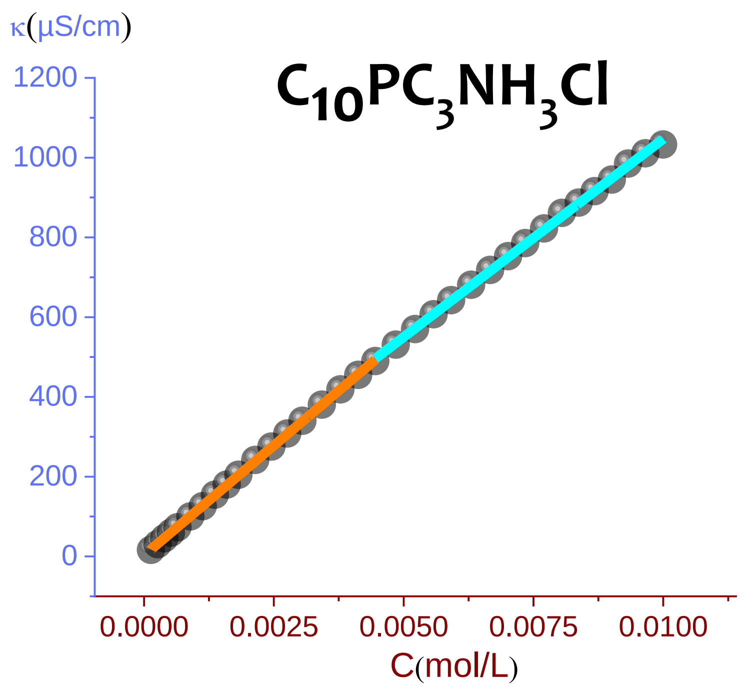

Figure.S32 Specific conductivity (κ) as a function of concentration of $C_{10}PC_3NH_3Cl$

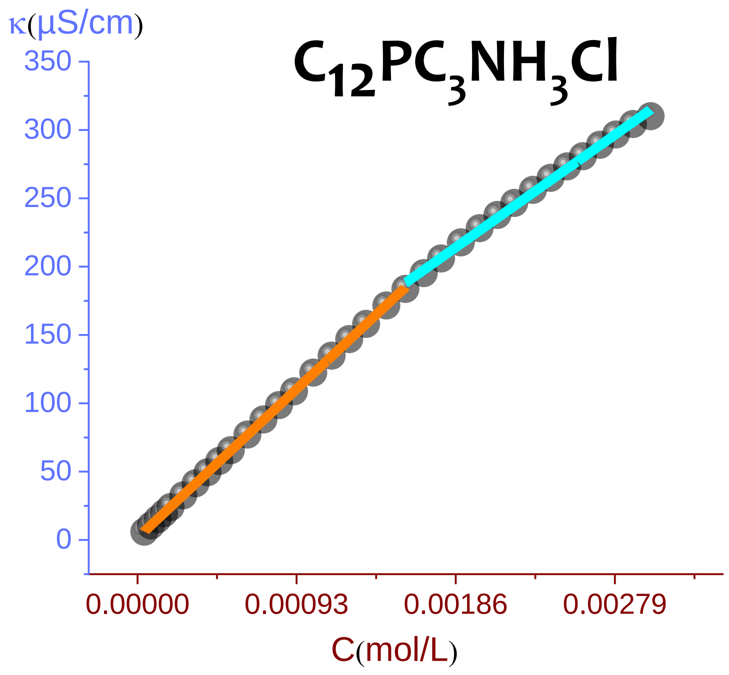

Figure.S33 Specific conductivity (κ) as a function of concentration of $C_{12}PC_3NH_3Cl$

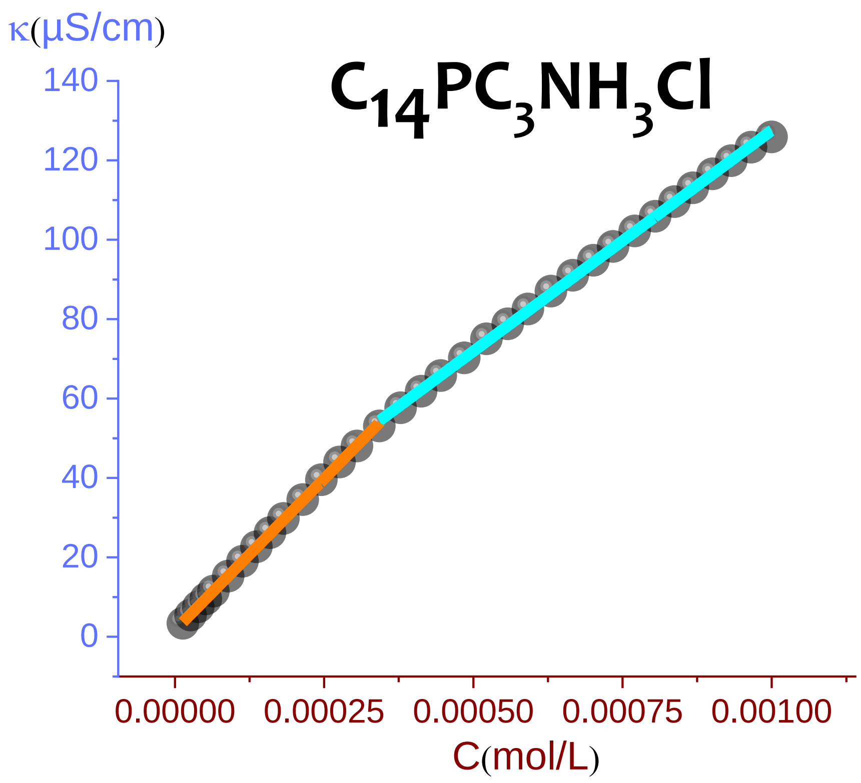

Figure.S34 Specific conductivity (κ) as a function of concentration of $C_{14}PC_3NH_3Cl$

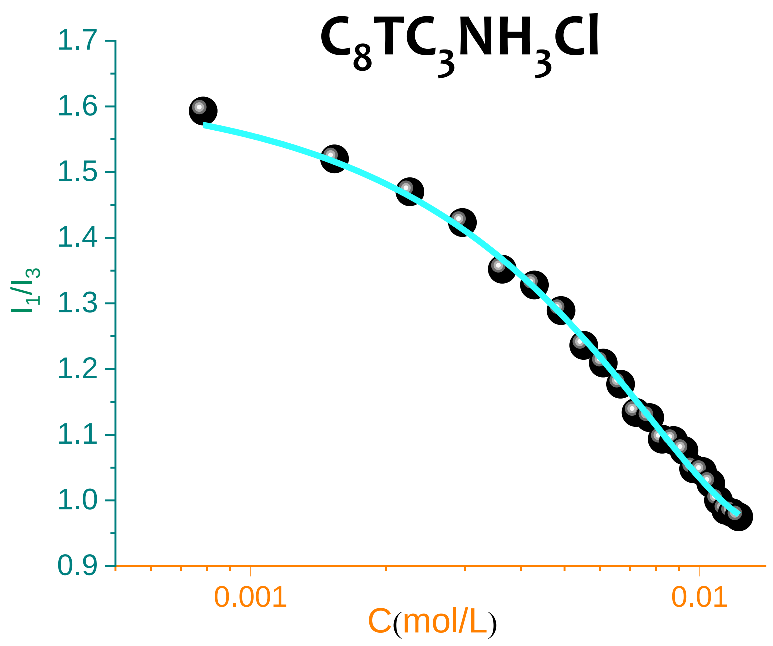

Figure.S35 Variation of I_1/I_3 ratio of pyrene fluorescence as a function of concentration $C_8TC_3NH_3Cl$

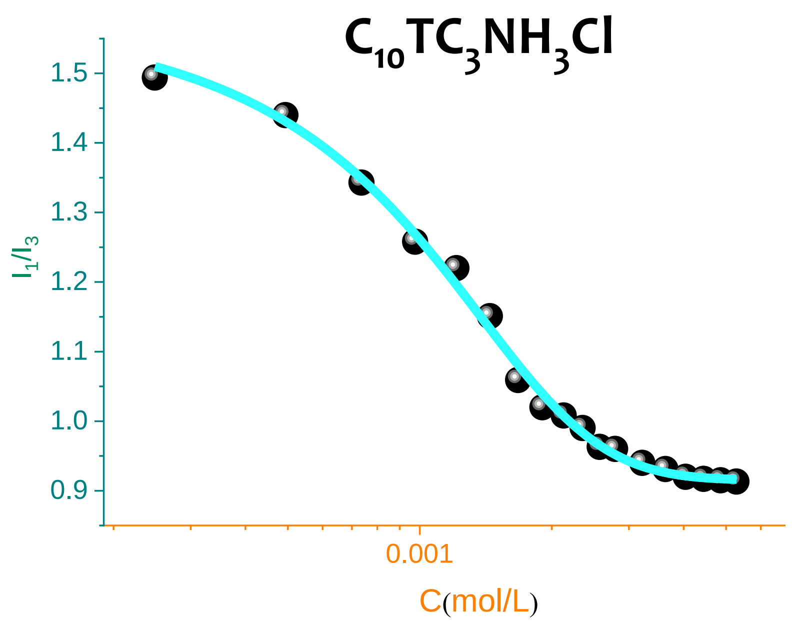

Figure.S36 Variation of I_1/I_3 ratio of pyrene fluorescence as a function of concentration $C_{10}TC_3NH_3Cl$

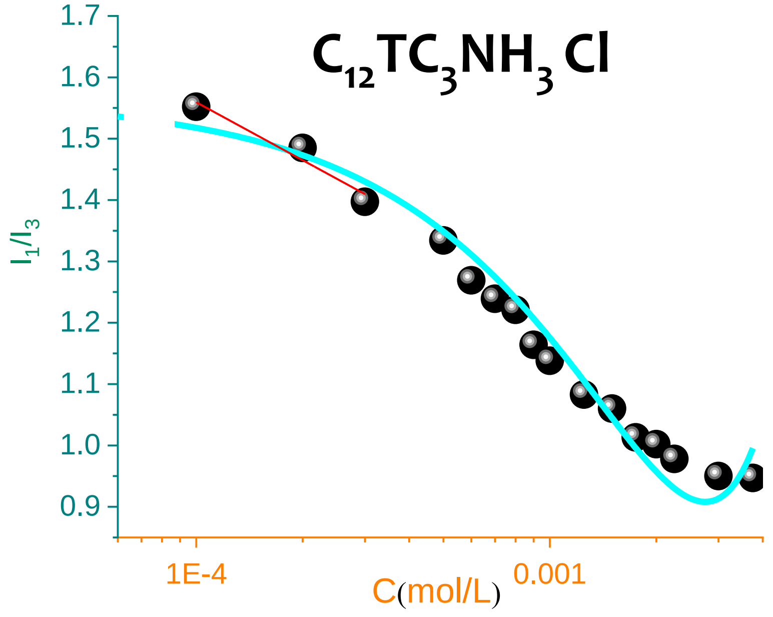

Figure.S37 Variation of I_1/I_3 ratio of pyrene fluorescence as a function of concentration$C_{12}TC_3NH_3Cl$

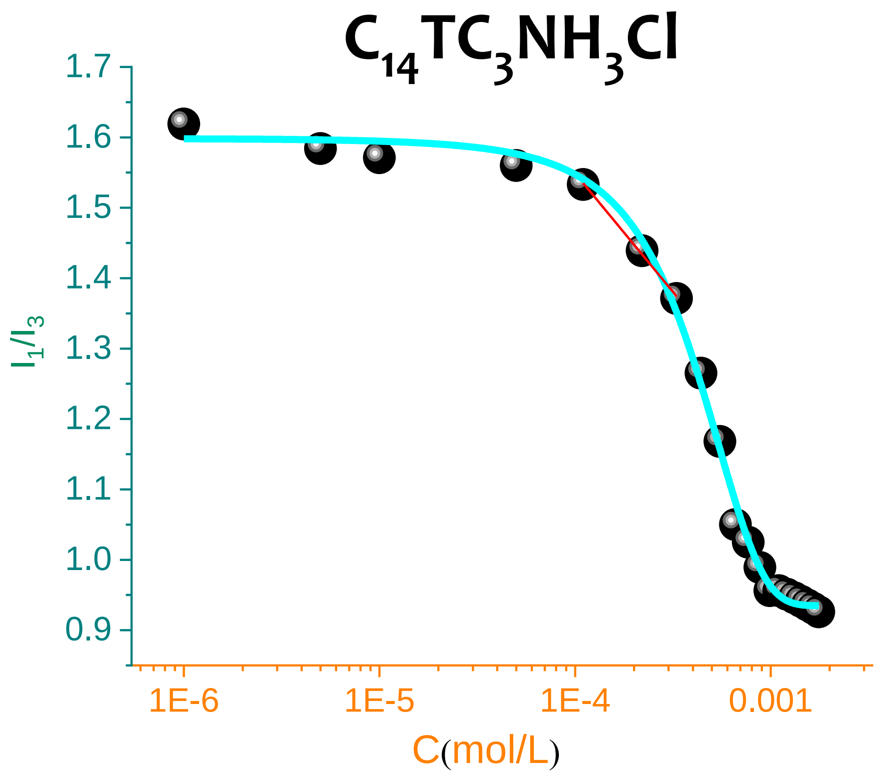

Figure.S38 Variation of I_1/I_3 ratio of pyrene fluorescence as a function of concentration$C_{14}TC_3NH_3Cl$

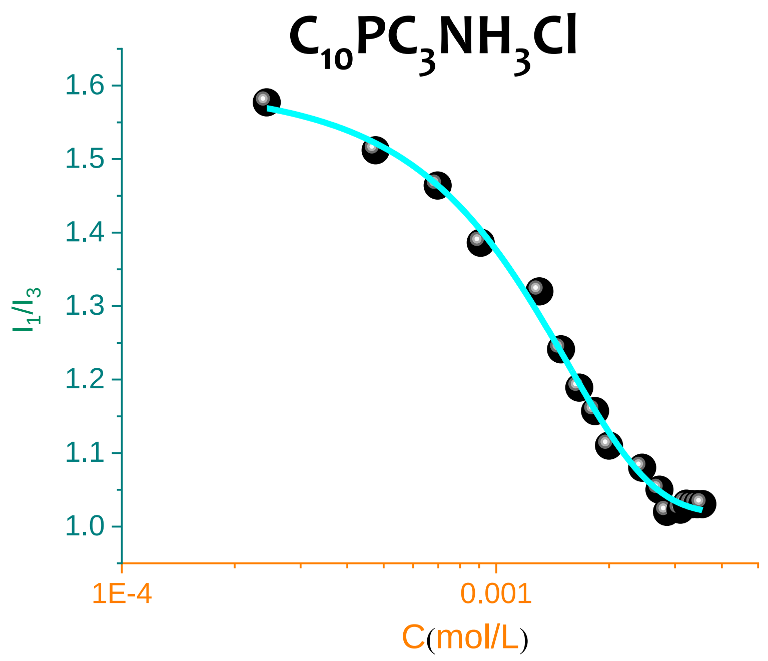

Figure.S39 Variation of I_1/I_3 ratio of pyrene fluorescence as a function of concentration$C_{14}PC_3NH_3Cl$

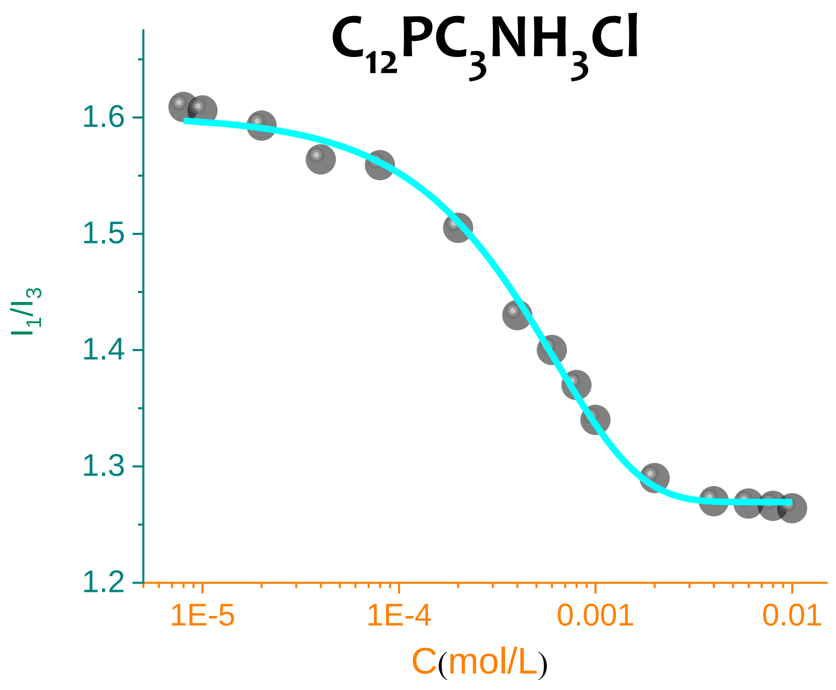

Figure.S40 Variation of I_1/I_3 ratio of pyrene fluorescence as a function of concentration$C_{12}PC_3NH_3Cl$

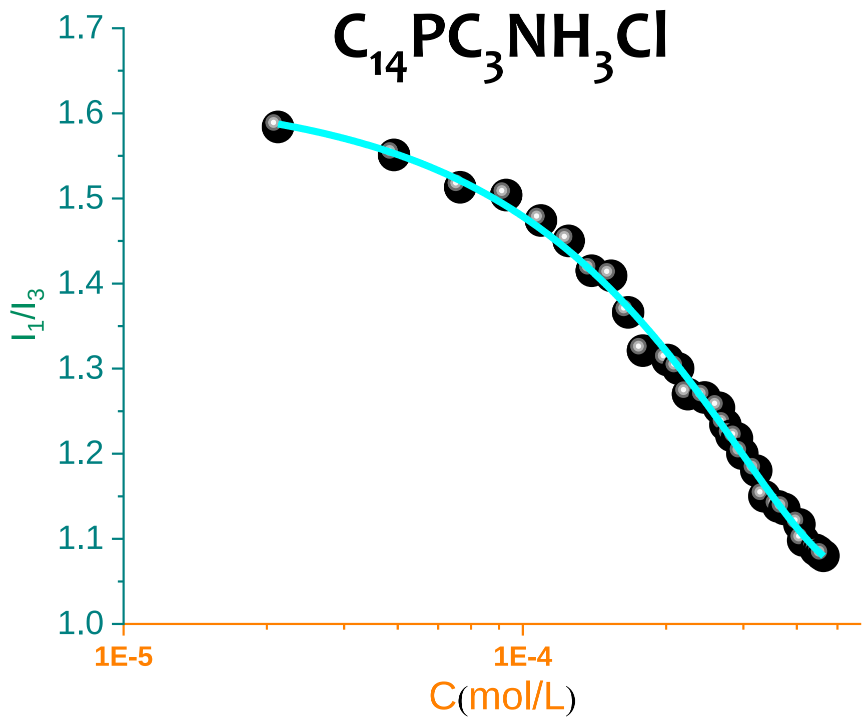

Figure.S41 Variation of I_1/I_3 ratio of pyrene fluorescence as a function of concentration$C_{14}PC_3NH_3Cl$

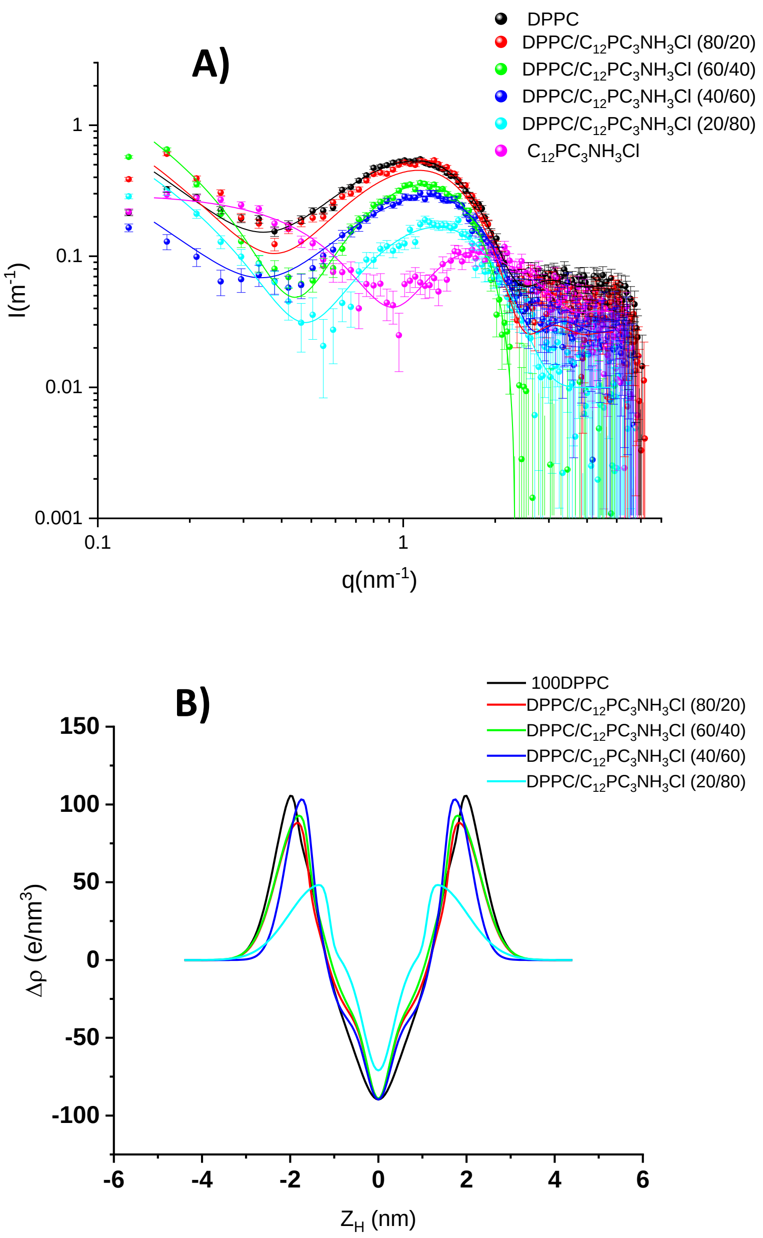

Figure.S42 A) Scattered intensity patterns as a function of scattering vector modulus for DPPC and $C_{12}PC_3NH_3Cl$ and their mixtures the curves correspond to the best fit of Gaussian bilayers or core-shell models. B) The corresponding electron density profiles of the bilayer models corresponding to the best fits of A).

Table S1 Fitting parameters of Gaussian bilayers for DPPC/$C_{12}PC_3NH_3Cl$ mixtures.

	DPPC/$C_{12}PC_3NH_3Cl$				
	DPPC	80/20	60/40	40/60	20/80
χ^2_{red}	1.19	0.75	1.24	1.02	1.05
**σ_h(nm)**	0.47±0.05	0.50±0.05	0.52±0.05	0.38±0.05	0.71±0.05
**ρ_h(e/nm^3)**	107±10	87±10	92±10	103±10	47±5
**Z_h(nm)**	1.87±0.05	1.81±0.05	1.77±0.05	1.72±0.05	1.30±0.05
**σ_c (nm)**	0.43±0.10	0.26±0.10	0.26±0.10	0.28±0.10	0.31±0.10

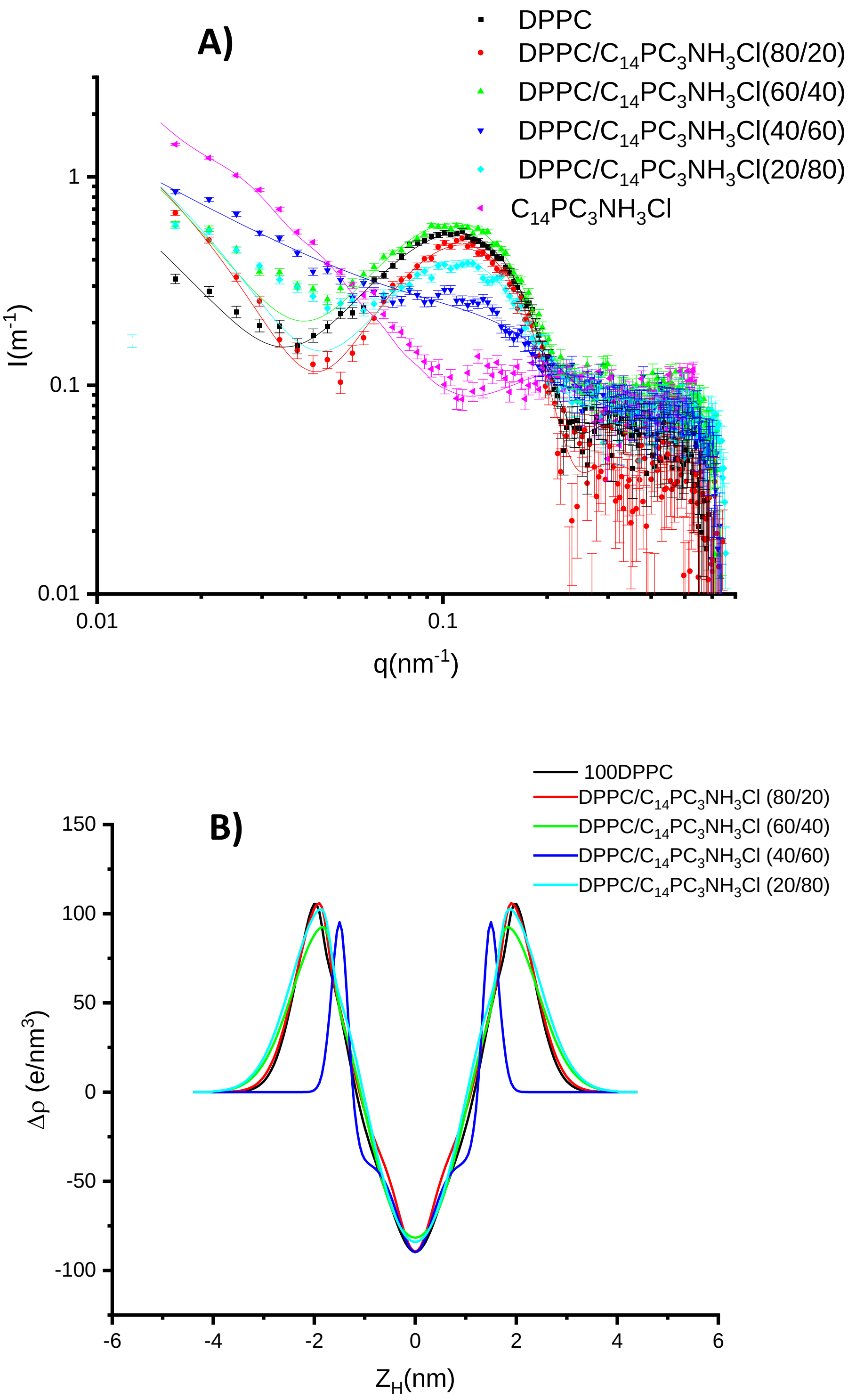

Figure.S43 A) Scattered intensity patterns as a function of scattering vector modulus for DPPC and $C_{14}PC_3NH_3Cl$ and their mixtures the curves correspond to the best fit of Gaussian bilayers or core-shell models. B) The corresponding electron density profiles of the bilayer models corresponding to the best fits of A).

Table S2 Fitting parameters of Gaussian bilayers for DPPC/$C_{14}PC_3NH_3Cl$ mixtures.

	DPPC/$C_{14}PC_3NH_3Cl$				
	DPPC	80/20	60/40	40/60	20/80
χ^2_{red}	1.19	2.12	4.7	2.55	6.1
**σ_h(nm)**	0.47±0.01	0.52±0.01	0.67±0.02	0.17±0.01	0.66±0.03
**ρ_h(e/nm^3)**	107±10	107±13	103±15	99±12	105±15
**Z_h(nm)**	1.87±0.05	1.84±0.05	1.71±0.06	1.47±0.05	1.78±0.08
**σ_c (nm)**	0.43±0.05	0.31±0.05	0.99±0.10	0.35±0.05	0.69±0.10

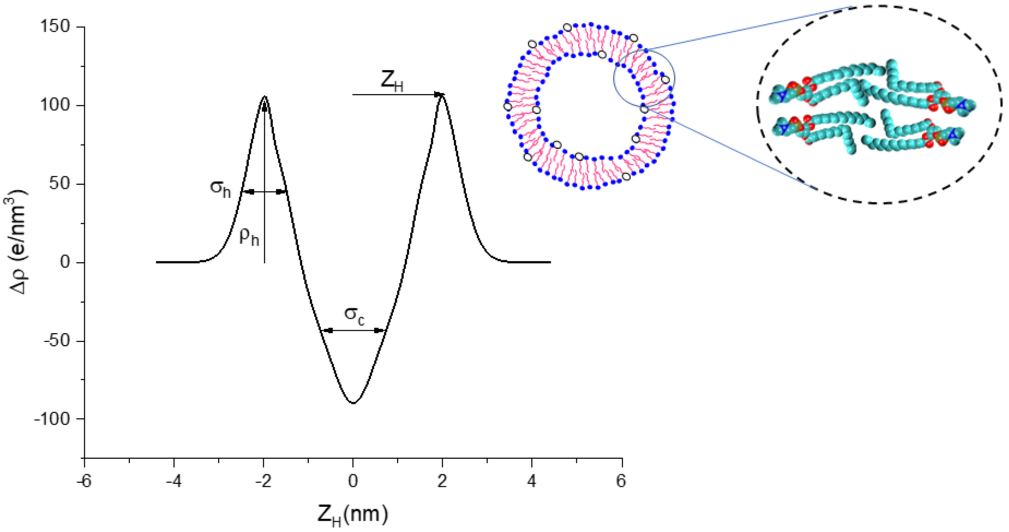

Figure.S44. Gaussian vesicle model

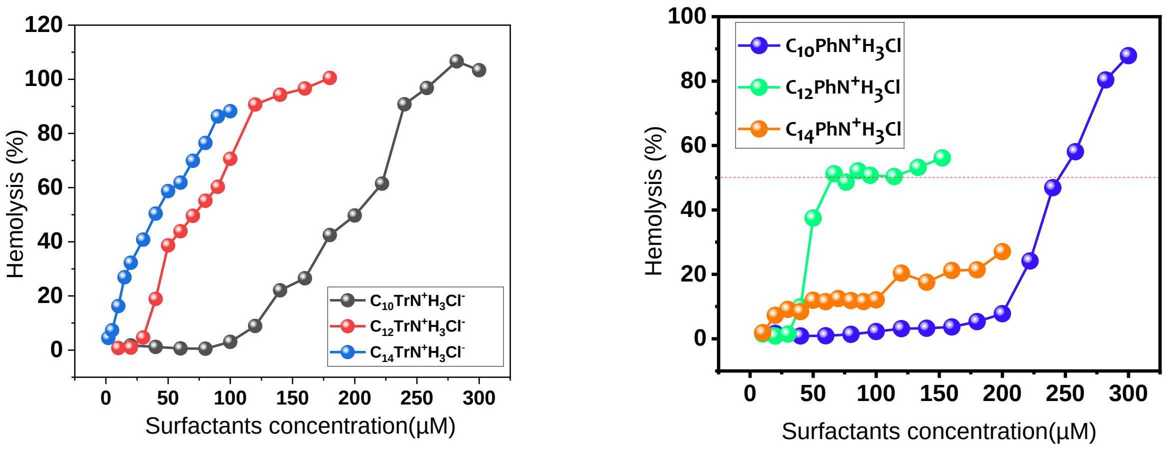

Figure. S45. Hemolysis (%) as a function of surfactants concentration.

Table S3 Hemolytic activity of tryptophan and phenylalanine-based surfactants.

	Hemolysis (HC_{50}) in µM
$C_{10}TC_3NH_3Cl$	200.51
$C_{12}TC_3NH_3Cl$	69.88
$C_{14}TC_3NH_3Cl$	40.27
$C_{10}PC_3NH_3Cl$	244.88
$C_{12}PC_3NH_3Cl$	64.55
$C_{14}PC_3NH_3Cl$	Not Hemolytic

Table S4 Results of the interaction details and docking score in (kcal/mol) of CnBenzalkonium derivative (From C_8 to C_{14} carbon atoms) against the peptidoglycan glycosyltransferase (PDB ID:2OQO).

Inhibitors	Score Kcal/mol	Ligand	Receptor	Interactions		Distance Å
				Categories	Types	
**C_8Benzalkonium**	-5.9	Ring(C6)	ARG225	Hydrophobic	Pi-Alkyl	5.0856
**C_{10}Benzalkonium**	-5.8	Ring(C6)	ALA141	Hydrophobic	Pi-Sigma	3.52615
		Ring(C6)	ILE93	Hydrophobic	Pi-Alkyl	5.3345
		Ring(C6)	ILE138	Hydrophobic	Pi-Alkyl	5.14622
		Ring(C6)	LEU142	Hydrophobic	Pi-Alkyl	5.4331
**C_{12}Benzalkonium**	-7.2	C(CH2)	GLY114	Hydrogen Bond	Carbon Hydrogen Bond	2.82665
		C(CH2)	GLN113	Hydrogen Bond	Carbon Hydrogen Bond	3.39365
		C(CH3)	THR82	Hydrogen Bond	Carbon Hydrogen Bond	3.34222
		C(CH3)	GLU83	Hydrogen Bond	Carbon Hydrogen Bond	2.99631
		Ring(C6)	ASP84	Hydrophobic	Pi-Sigma	3.91375
		Ring(C6)	ALA97	Hydrophobic	Pi-Sigma	3.50131
**C_{14}Benzalkonium**	-7.4	C(CH2)	GLY114	Hydrogen Bond	Carbon Hydrogen Bond	2.77969
		C(CH2)	GLN113	Hydrogen Bond	Carbon Hydrogen Bond	3.37694
		C(CH3)	THR82	Hydrogen Bond	Carbon Hydrogen Bond	3.28951
		C(CH3)	GLU83	Hydrogen Bond	Carbon Hydrogen Bond	3.02791
		Ring(C6)	ASP84	Hydrophobic	Pi-Sigma	3.91929
		Ring(C6)	ALA97	Hydrophobic	Pi-Sigma	3.51456

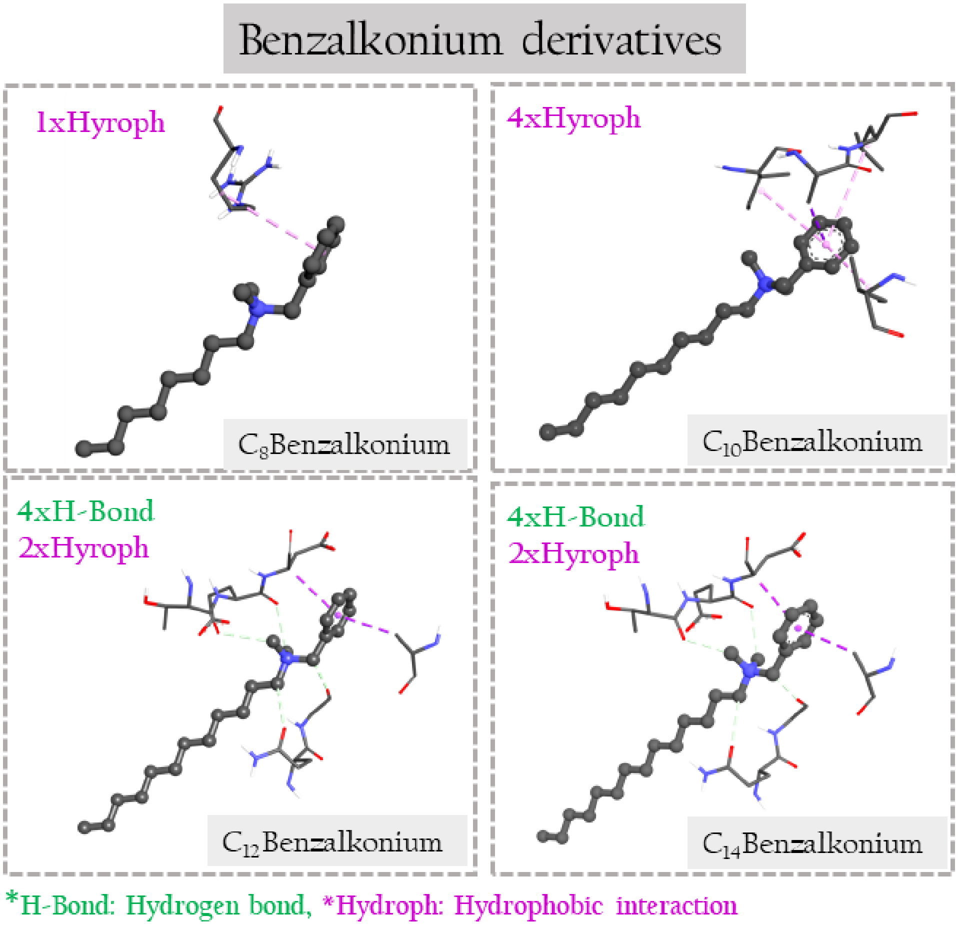

Figure S46: Three-dimensional (3 D) closest interactions between active site residues of peptidoglycan glycosyltransferase (PDB ID:2OQO) With CnBenzalkonium (From C_8 to C_{14} carbon atoms) derivatives
